# Protective Effects of a New Human Placental Extract Against Hair Graying and Chemotherapy-Induced Peripheral Neuropathy

**DOI:** 10.3390/ijms27104188

**Published:** 2026-05-08

**Authors:** Eri Horio, Yasuhiro Katahira, Natsuki Yamaguchi, Miki Igarashi, Hideaki Hasegawa, Satomi Miyakawa, Shota Toda, Izuru Mizoguchi, Ning Qu, Hiromitsu Anamizu, Shinichiro Ikeda, Hirohiko Matsumoto, Takayuki Yoshimoto

**Affiliations:** 1 Department of Immunoregulation, Institute of Medical Science, Tokyo Medical University, 6-1-1 Shinjuku, Shinjuku-ku, Tokyo 160-8402, Japan; 2 Advanced Medical Tokyo Corporation, 2-6-1 Nihonbashi-Honcho, Chuo-ku, Tokyo 103-0023, Japan; 3 Japanese Society of Umbilical Cord and Placenta Medicine, 4-1 Kofuna-cho, Nihonbashi, Chuo-ku, Tokyo 103-0024, Japan

**Keywords:** antioxidation, chemotherapy-induced peripheral neuropathy, hair graying, human placental extract, proteomic analysis, neurite outgrowth

## Abstract

As stem cell therapy in regenerative medicine becomes more socially accepted, human perinatal tissue is attracting attention as a source because it can be harvested non-invasively. Human placental extracts (HPEs), which are prepared using acid hydrolysis and autoclaving, have been approved for treating menopausal disorders and liver dysfunction. This study investigated a new HPE formulation prepared under milder conditions by omitting acid hydrolysis and autoclaving to improve its effectiveness. The new HPE contains relatively high-molecular-weight proteins, including high levels of thioredoxin-1, as well as primarily extracellular matrix proteins such as thrombospondin-1. These proteins appear to be intact or partially fragmented, but they can still potentially maintain their domain structure. The HPE showed both antioxidant and neurite outgrowth activities in a neuronal cell line SH-SY5Y. In a mouse model of hair graying caused by X-ray irradiation, multiple administration of the HPE significantly reduced it. Additionally, the HPE, but not heat-inactivated HPE, alleviated the mechanical allodynia in a mouse model with chemotherapy-induced peripheral neuropathy. Due to the fact that HPE can be produced non-invasively in large quantities in a short time without the need for culturing, the new HPE may have the potential to be an effective and feasible therapy via multiple mechanisms.

## 1. Introduction

In terms of advancing the social implementation of regenerative medicine using stem cells, there has been a great deal of attention focused on human perinatal appendages, such as the placenta, umbilical cord, and amniotic and chorionic membranes, as they are a rich source of multipotent cells, cytokines and growth factors. This is because they are generally disposed of as medical waste under normal delivery practices without any additional invasive processes being required to harvest them. The placenta is a unique temporary organ that facilitates the coexistence of the immunologically different organisms of the mother and the fetus and also promotes fetal growth and development by supplying nutrition and removing metabolites [1,2]. Human placental extracts (HPEs), Melsmon and Laennec, prepared by acid hydrolysis and proteolysis using enzymes such as pepsin, followed by sterilization by autoclaving, were approved for the treatment of menopausal, lactational, and liver dysfunction disorders in Japan in the 1950s~1970s [3,4]. The main active components of HPEs are low-molecular-weight substances, including amino acids, peptides, vitamins, steroid hormones, sugars and nucleic acids, but not proteins [5,6].

Since then, various HPEs have been prepared using different methods of decellularization, extraction and sterilization. An increasing amount of research has shown that HPEs have anti-inflammatory, antioxidant, anti-allergic, analgesic and tissue regenerative properties in various experimental settings involving autoimmune and inflammatory diseases, osteoarthritis, ischemic brain damage, liver damage, wound healing and diabetes [2,7]. However, most of the literature [5,8] does not describe the protocols for preparing HPEs in detail. In addition, due to the severe conditions used for HPE preparation, few reports have performed comprehensive analyses of HPE protein composition, such as proteomic or antibody array analysis.

Recently, EpiFix, a placental tissue allograft consisting solely of dehydrated human amnion and chorion membrane (dHACM), which is prepared without acid hydrolysis or autoclaving, was approved as a medical device in Japan in 2021 for the treatment of diabetic foot ulcers and venous leg ulcers [9,10]. This preparation process involves gently cleansing the amnion and chorion layers and laminating them to form the dehydrated graft under controlled drying conditions. All donors are tested to ensure they are free of several infectious diseases. The resulting membrane likely retains the native composition of the extracellular matrix and sustains the gradual release of bioactive growth factors. It accelerates tissue wound healing and cell growth and is an off-the-shelf product with a shelf life of five years [11].

In this study, to improve the effectiveness of HPE, we prepared a new formulation from a mixture of umbilical cord, placenta and amnion under milder conditions, omitting acid hydrolysis and autoclaving. We then performed biochemical and biological functional analyses. First, electrophoresis, followed by protein staining, revealed that the HPE contains proteins with a relatively wide range of molecular weights, including those with higher molecular weights. Comprehensive analyses of a protein composition of the HPE using data-independent acquisition (DIA) proteomics revealed the dominant presence of extracellular matrix proteins, including collagen, fibronectin, laminin and thrombospondin-1 (TSP-1) [12,13,14], as well as cytokines, growth factors and other molecules including high levels of the antioxidant molecule thioredoxin-1 (TRX-1) [15,16,17]. The HPE administration reduced hair graying caused by X-ray irradiation and alleviated chemotherapy-induced peripheral neuropathy (CIPN) with paclitaxel (PTX) in mice, possibly through its antioxidant and neurite outgrowth activities. To the best of our knowledge, this is the first report on the therapeutic effects of HPE against hair graying and CIPN.

## 2. Results

### 2.1. New HPE Contains a Variety of Proteins Covering a Wide Range of Molecular Weights Mainly Including Extracellular Matrix Proteins

Compared with the protocols used for preparing most HPEs, including Melsmon and Laennec [6], a new HPE formulation was prepared from a mixture of human umbilical cord, placenta, and amnion under milder conditions, in which harsher processes, such as hydrochloric acid hydrolysis at high temperature and sterilization by pressure steam (autoclaving), were omitted. The cryopreserved tissues confirmed to be negative for viral and bacterial infections by serological testing were used and proteolyzed with pepsin to liberate proteins from tissues under strongly acidic conditions. It enzymatically cleaves peptide bonds and breaks down complex protein structures into more soluble peptides, thereby facilitating their efficient extraction. Pepsin is useful because it is active only under strongly acidic conditions and is inactive under neutral conditions. Then, the extract was sterilized by filtration through 0.45 and 0.2 µm filters and lyophilized.

**Figure 1:**
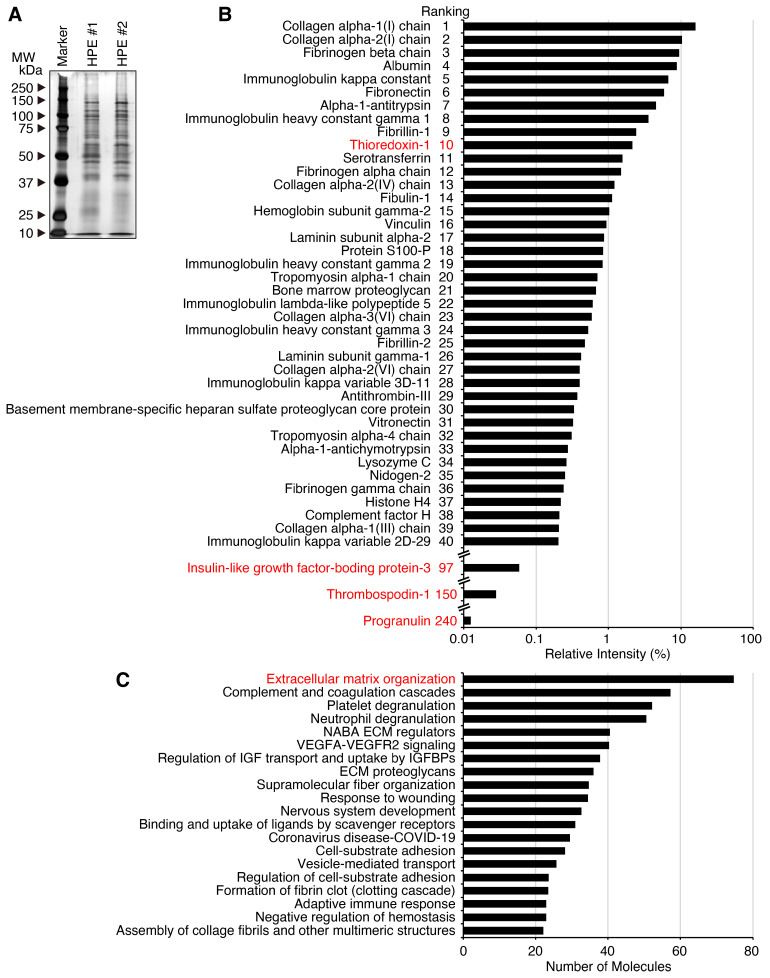
New HPE contains a variety of proteins covering a wide range of molecular weights mainly including extracellular matrix proteins. (**A**) The HPEs from two different lots (#1 and #2) were subjected to SDS-PAGE and protein analysis with silver staining, which detected a variety of proteins covering a wide range of molecular weights. (**B**) DIA proteomic analysis revealed that the HPE (lot #1) contains 875 proteins (Supplementary Table S1), the majority of which are extracellular matrix proteins. The top 40 of these proteins together with IGFBP-3, TSP-1 and PGRN are shown. (**C**) The results of the DIA proteomic analysis were subjected to a gene ontology analysis, which revealed that the HPE contains proteins predominantly related to extracellular matrix organization. A full-length gel (**A**) is shown in Figure S1A.

Using the new HPE, we performed biochemical and functional analyses. First, SDS-PAGE analysis of two different lots of the HPE, followed by silver staining, revealed an abundance of proteins with a relatively wide range of molecular weights, including higher-molecular-weight proteins in both lots (Figure 1A). The patterns of the detected protein bands are highly similar in these two lots. DIA proteomic analysis was performed to further investigate the individual molecules and detected 875 protein molecules (Table S1). The analysis revealed a high content of extracellular matrix proteins, including collagen, fibrinogen, albumin, immunoglobulin, fibronectin, α-1-antitrypsin and fibrillin (Figure 1B). Gene ontology analysis revealed that the top-ranked molecules are proteins related to the organization of the extracellular matrix, followed by the complement and coagulation cascades and platelet degranulation (Figure 1C). Several functionally interesting molecules were detected among the proteins: TRX-1 [15,16,17], an antioxidant with a ranking of 10; insulin-like growth factor binding protein (IGFBP)-3 [18], a growth inhibitor with a ranking of 97; TSP-1 [12,13,14], an inducer of synaptogenesis with a ranking of 150; and progranulin (PGRN) [19], a neuroprotectant with a ranking of 240.

These results suggest that the new HPE contains a variety of proteins with a wide range of molecular weights, primarily extracellular matrix proteins.

### 2.2. New HPE Contains Intact and Partially Fragmented High-Molecular-Weight Proteins

**Figure 2:**
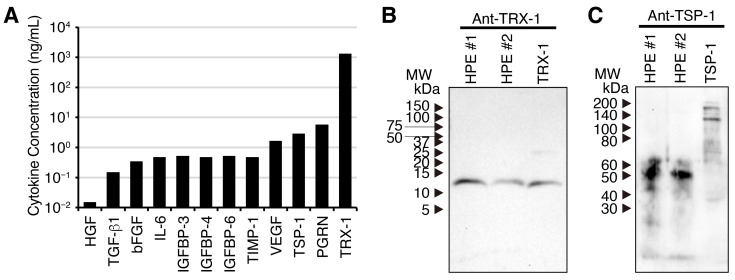
New HPE contains intact and partially fragmented high-molecular-weight proteins. (**A**) The HPE (lot #1) was subjected to ELISA analysis to determine the concentrations of proteins present. The HPEs from two different lots (#1 and #2) were subjected to Western blot analysis following SDS-PAGE, using antibodies against TRX-1 (**B**) and TSP-1 (**C**). The anti-TRX-1 antibody detected a single band around 12 kDa that is a similar molecular weight to recombinant TRX-1, suggesting that it is intact (**B**). In contrast, the anti-TSP-1 antibody detected bands with a much lower molecular weight around 50 kDa than recombinant TSP-1, whose intact molecular weight is 170 kDa, suggesting that it had been partially fragmented during preparation (**C**). Full-length blots (**B**,**C**) are shown in Figure S1B,C.

To confirm the presence and concentrations of the detected proteins, sandwich ELISAs were performed that were specific to individual cytokines (Figure 2A). The ELISA analysis revealed that the abundance detected by DIA proteomic analysis slightly differs from that detected by the individual cytokine-specific ELISA. Indeed, TRX-1, IGFBP-3, TSP-1 and PGRN were also detected using specific sandwich ELISAs. Because the protocol for preparing the HPE involves proteinase digestion with pepsin to release cytokines and growth factors from placental tissues, this suggests the possibility of fragmentation of the released cytokines and growth factors. To examine this possibility, Western blot analysis was performed following SDS-PAGE of the HPE, using specific antibodies against TRX-1 and TSP-1. The anti-TRX-1 antibody detected similar bands at around 12 kDa in human recombinant TRX-1 and two lots of the HPE. This suggests that the TRX-1 in both HPE lots is intact and has not fragmented (Figure 2B). By contrast, the anti-TSP-1 antibody detected a 170 kDa band alongside the lower, potentially degraded band in intact TSP-1. However, it only detected a much lower 50 kDa band in both HPE lots and did not detect the 170 kDa band (Figure 2C). Given the pepsin treatment used to prepare the HPE, it is highly likely that the band detected is one of the fragmented forms of TSP-1.

These results suggest that the HPE contains intact and partially fragmented, yet still high-molecular-weight, proteins.

### 2.3. New HPE Has Potent Antioxidant Activity in Human Neuronal Cell Line SH-SY5Y

**Figure 3:**
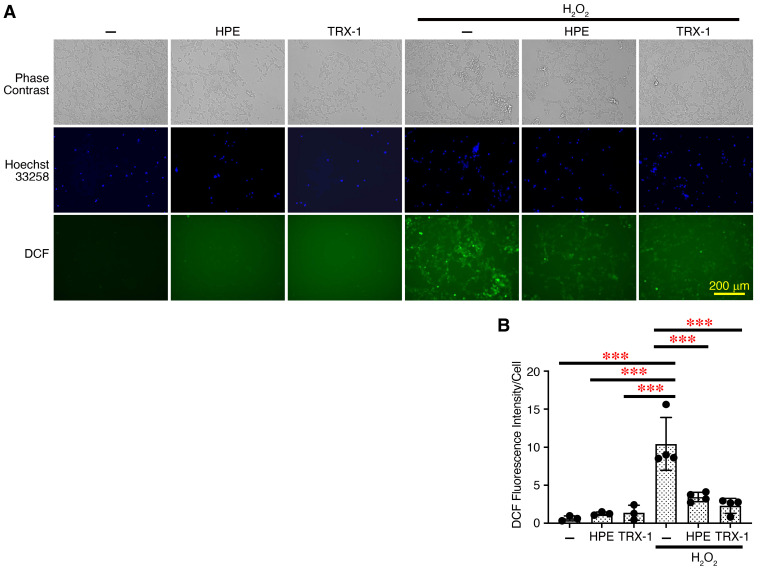
New HPE has potent antioxidant activity in human neuronal cell line SH-SY5Y. (**A**) SH-SY5Y cells were preincubated with HPE (lot #2, 1%) or TRX-1 (1 µg/mL) for 3 h. These cells were then treated with DCFDA, stimulated with H_2_O_2_ (50 μM) for 1 h, and counterstained with Hoechst 33258. Representative photographs of the ROS generation are shown. Larger images are shown in Figure S2. (**B**) The fluorescence intensity of DCF and Hoechst 33258 was quantified using Fiji software and calculated as arbitrary unit per cell. Data are shown as the mean ± SD (*n* = 4) and are representative of three independent experiments. *p* values were determined by one-way analysis of variance with Tukey’s multiple comparisons test. *** *p* < 0.001.

Thus, the HPE contains intact proteins, but some of the proteins in the HPE appeared to be partially fragmented due to pepsin treatment. However, extracellular matrix proteins generally have a higher molecular weight and several functional domains, each with its own biological activity [20,21]. Previously, HPE has been reported to possess antioxidant and neurite outgrowth activities [5,22]. Therefore, we next investigated the biological activities of the new HPE in vitro. First, we explored the antioxidant activity using the human neuroblastoma cell line SH-SY5Y, which has been frequently used as a human neuronal model cell line. The effect of the HPE on the reactive oxygen species (ROS) generation induced by H_2_O_2_ was examined. The addition of H_2_O_2_ greatly induced ROS generation by increasing 2′,7′-dichlorofluorescein (DCF)^+^ cells (Figure 3A,B). However, preincubation of the cells with the HPE significantly reduced the intensity of DCF^+^ cells. Similarly, the preincubation with TRX-1, which is known to have potent antioxidant activity [17], also significantly reduced it.

Thus, the HPE has potent antioxidant activity in SH-SY5Y cells as TRX-1 has.

### 2.4. New HPE Induces Neurite Outgrowth of Human Neuronal Cell Line SH-SY5Y

**Figure 4:**
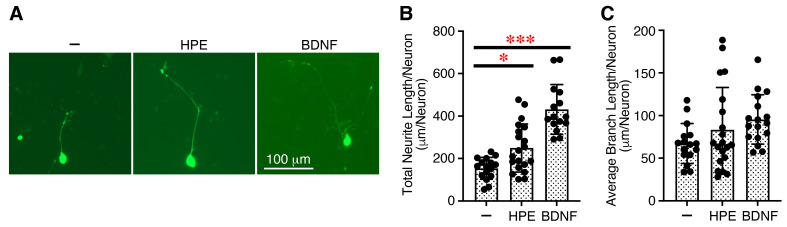
New HPE increases neurite outgrowth of human neuronal cell line SH-SY5Y. SH-SY5Y cells in DMEM/F-12 medium containing FBS (10%) were first transiently transfected with the pGFP plasmid. After 24 h, the cells were stimulated with ATRA (10 µM) in DMEM/F-12 medium containing FBS (2%). After 3 days, the medium was changed to DMEM/F-12 medium containing ATRA (10 µM) and FBS (1%) with and without HPE (lot #2, 5%) or BDNF (50 ng/mL) as a positive control and cultured for an additional 3 days. Representative images are shown (**A**), and total neurite length (**B**) and average branch length (**C**) per neuron of GFP^+^ neurite-bearing cells were measured using Fiji software. Data are shown as the mean ± SD (*n* = 17~20 neurons of three combined independent experiments). *p* values were determined by one-way analysis of variance with the Tukey’s multiple comparisons test. * *p* < 0.05, *** *p* < 0.001.

Human neuronal cell line SH-SY5Y is widely used as an in vitro model of neurodegeneration, neurotrauma, developmental neurotoxicity, and neurite outgrowth [23,24]. It has been reported that TSP-1 promotes process outgrowth in neurons from the peripheral and central nervous systems [25] and mediates axon regeneration in retinal ganglion cells [26]. PGRN has also shown neurite outgrowth activity [27]. Since both are present in the HPE (Figure 1B), we next examined the effects of the HPE on the neurite outgrowth of SH-SY5Y cells that had been differentiated with all-trans-retinoic acid (ATRA). The HPE significantly increased the neurite length (Figure 4A,B) but only slightly tended to enhance the branch length, as brain-derived neurotrophic factor (BDNF), a positive control cytokine, did (Figure 4A,C).

These results suggest that the HPE acts on nerve cells and increases the neurite outgrowth of SH-SY5Y cells.

### 2.5. Administration of New HPE Reduces Hair Graying Caused by X-Ray Irradiation

**Figure 5:**
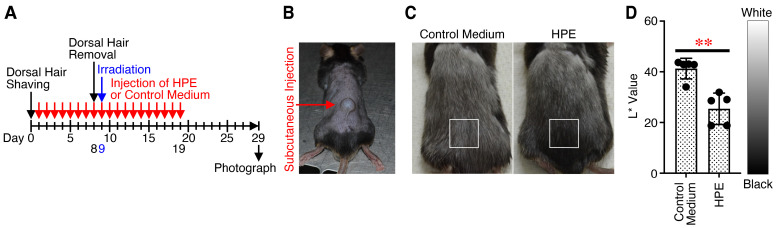
Administration of new HPE reduces hair graying caused by X-ray irradiation. (**A**) After removal of dorsal hair, HPE (lot #1, 10%) or control medium (100 µL) was subcutaneously administrated into the dorsal skin from day 1 every day for 19 days, and photographs of the dorsal area were taken with time. (**B**) A representative image of the dorsal skin just after the subcutaneous administration is shown. (**C**) Representative photographs of the dorsal area on day 29 are shown. (**D**) Hair blackness in the areas marked by white boxes (**C**) was assessed and quantified from each photograph with CIELAB color system using Fiji software. The L* value indicates hair brightness, from 0 (black) to 100 (white). The average L* value is shown as the result of the part of dorsal area where the administration was performed. Data are shown as the mean ± SD (*n* = 5) and are representative of two independent experiments. *p* values were determined using the unpaired two tailed Student’s *t*-test. ** *p* < 0.01.

To investigate the potential therapeutic effects of the HPE in vivo, we used two different disease mouse models; hair graying caused by X-ray irradiation and PTX-induced peripheral neuropathy. Hair graying is mainly induced by a decrease in the number of melanocyte stem cells, which is caused by an excess accumulation of oxidative stress [28,29]. Therefore, we first examined its therapeutic effect against hair graying caused by X-ray irradiation. The HPE or control medium was subcutaneously administered into the hair-removed dorsal skin every day (Figure 5A,B). On day 9, the mice were irradiated with X-ray after removal of hair via waxing on day 8. The color of the dorsal hair was observed and photographed with time. After X-ray irradiation, the hair color gradually turned black. The hair color of mice administered with the HPE was significantly blacker than that of mice administered with control medium, which was determined on day 29 (Figure 5C,D).

These results suggest that administration of the HPE reduces hair graying caused by X-ray irradiation, possibly via its antioxidant activity.

### 2.6. Administration of New HPE Alleviates PTX-Induced Peripheral Neuropathy in a Heat-Sensitive Manner

**Figure 6:**
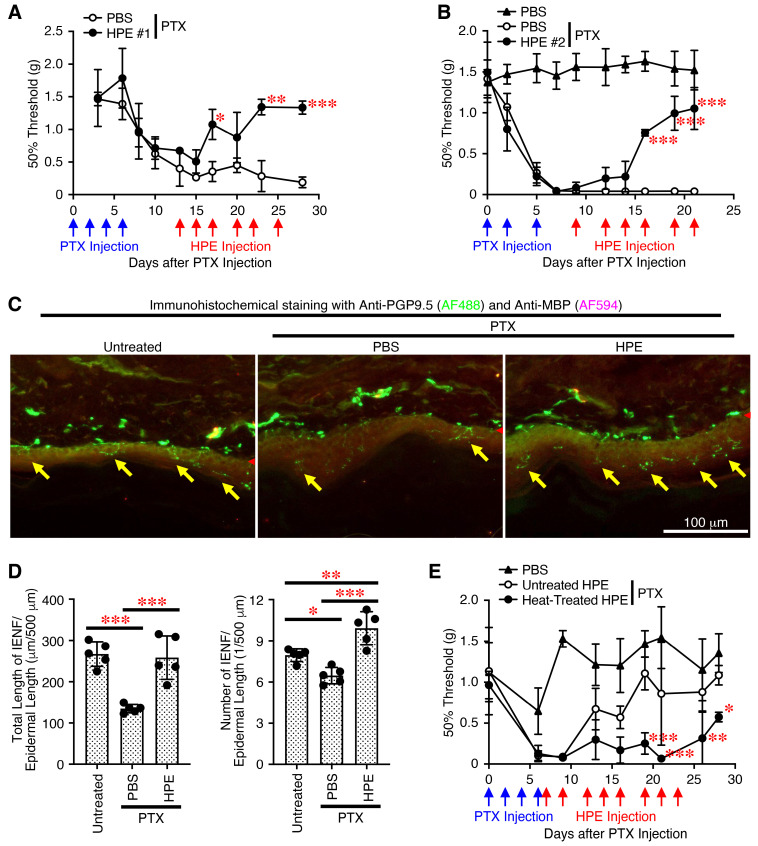
Administration of new HPE alleviates PTX-induced mechanical allodynia in a heat-sensitive manner. PTX was administered to C57BL/6 mice intraperitoneally at two-day intervals for a total of three to four administrations. Around 10 days later, HPE (10%) lots #1 (**A**), #2 (**B**–**E**) or PBS were administered to the mice intraperitoneally multiple times, as indicated. PBS was also administered to PTX-untreated mice. Mechanical allodynia was assessed for the hind paw to withdraw in response to stimulation with von Frey filaments. The 50% von Frey threshold was calculated from the response pattern. (**C**) On day 32, tissue samples from the skin on the plantar surface of the hind paws were analyzed using immunohistochemistry to detect PGP9.5^+^ IENFs (indicated by yellow arrows) in the epidermis and MBP^+^ Schwann cells in the dermis. The skin basement membrane was indicated by red arrowheads. Representative images are shown. (**D**) The length and number of PGP9.5^+^ IENFs per the epidermal length were determined. (**E**) The HPE was incubated at 95 °C for 30 min and then cooled immediately to 4 °C. PTX was administered to C57BL/6J mice intraperitoneally at two-day intervals for a total of four administrations. On day 7, untreated HPE and heat-inactivated HPE (10%) was administered multiple times to the mice, and PBS was also administered to PTX-untreated mice. Mechanical allodynia was then assessed. Data are shown as the mean ± SD (*n* = 5) and are representative of more than two independent experiments. *p* values were determined using one-way (**D**) or two-way analysis (**A**,**B**,**E**) of variance with a Tukey’s multiple comparisons test. * *p* < 0.05, ** *p* < 0.01, *** *p* < 0.001.

Several molecular mechanisms have been reported to alleviate the peripheral neuropathy caused by PTX, including the antioxidant activity and promotion of neurite outgrowth in neurons [30,31,32]. We finally examined the therapeutic effect of the HPE against PTX-induced peripheral neuropathy. Following repeated PTX administrations, the mice began to exhibit signs of mechanical allodynia, resulting in a decreased 50% threshold. Then, repeated administrations of the HPE gradually reversed the decrease in the 50% threshold, eventually restoring it to the original level, whereas repeated administrations of the control phosphate-buffered saline (PBS) did not (Figure 6A). A different lot of the HPE exhibited similar therapeutic effects against mechanical allodynia (Figure 6B).

The use of PTX in chemotherapy has been reported to retract peripheral nerve endings and reduce the number of intraepidermal nerve fibers (IENFs) in cancer patients and mouse models due to neuroinflammation, oxidative stress and mitochondrial dysfunction [30,33]. In addition to fiber loss, surviving IENFs become hyperexcitable and show abnormal spontaneous activity, leading to pain hypersensitivity, such as mechanical allodynia and thermal hyperalgesia. We then examined the effect of the HPE on the IENF length and number in the skin on the plantar surface of the hind paws of the mice. Immunohistochemical analysis using an antibody against protein gene product 9.5 (PGP9.5), a marker for IENFs [34], revealed that the total length and number of PGP9.5^+^ IENFs decreased following PTX administration but increased significantly following the administration of the HPE compared with PBS (Figure 6C,D).

To further explore whether proteins in the HPE contribute to alleviating PTX-induced peripheral neuropathy, we prepared the heat-inactivated HPE by incubating at 95 °C for 30 min and compared it to the untreated intact HPE. While the untreated HPE exhibited activity that alleviated PTX-induced mechanical allodynia, the heat-inactivated HPE lost most of its ability to do so (Figure 6E).

These results suggest that administration of the HPE alleviates PTX-induced peripheral neuropathy in a heat-sensitive manner, possibly via proteins.

## 3. Discussion

In this study, to improve the effectiveness of HPE, we prepared a new formulation from a mixture of umbilical cord, placenta and amnion under milder conditions, omitting acid hydrolysis and autoclaving used previously. Proteomics and ELISA analyses revealed that the HPE contains mainly extracellular matrix proteins, including collagen, fibronectin, laminin and TSP-1, as well as high levels of TRX-1. Although the HPE was prepared using pepsin, it contains intact and partially fragmented, yet high-molecular-weight, proteins. For instance, the TRX-1 remains intact, but the TSP-1 has partially fragmented into a smaller protein with a molecular weight of around 50 kDa. The HPE was demonstrated to exhibit a therapeutic effect against hair graying caused by X-ray irradiation. Additionally, the HPE showed a therapeutic effect against a PTX-induced CIPN mouse model, alleviating mechanical allodynia and restoring the IENFs. The mechanical allodynia-alleviating effect was reduced by the heat-inactivation of the HPE, which suggests that heat-sensitive proteins, such as cytokines and growth factors, are indeed involved. These effects are likely related to the antioxidant and neurite outgrowth activities of the HPE, as demonstrated using the SH-SY5Y neuronal cell line. These activities may be mediated by TRX-1, TSP-1 or PGRN, present in the HPE, because TRX-1 was reported to have antioxidant activity [35], and TSP-1 [36] and PGRN [27] were reported to have neurite outgrowth activity. However, further research is needed to prove the contribution of these proteins in the HPE to therapeutic activities.

There have been no detailed reports on the methods used to produce HPEs, including those approved as pharmaceuticals by the Japanese Ministry of Health, Labour and Welfare, such as Melsmon and Laennec. This is probably because the manufacturing method is a proprietary process, and its details are protected as a trade secret. Melsmon has been approved for treating menopausal disorders and milk secretion deficiency [3], and Laennec has been approved for treating cirrhosis of the liver [4]. However, a recent paper briefly describes and compares their manufacturing methods [6]. In the case of Melsmon, the frozen, infection-screened placentas are used after removing the umbilical cord and de-blooding the villous tissue. The material is hydrolyzed with hydrochloric acid at high temperature, neutralized to pH 7.0, and supplemented with benzyl alcohol. After adjusting component concentrations, the extract is ampouled and sterilized by high-pressure steam. In the case of Laennec, human placentas are refrigerated, washed, minced, homogenized with acetone, defatted, and dried. The dried material is acidified, proteolyzed with pepsin, and centrifuged. The precipitate is further hydrolyzed with hydrochloric acid at high temperature, filtered through activated charcoal, and combined with the supernatant. After removing hydrochloric acid using an anion exchange resin, the solution is adjusted to pH 6.1–6.4 and sterilized by high-pressure steam.

Currently, mesenchymal stem/stromal cells are sourced from umbilical cords, umbilical cord blood and amnions, but this requires cell culture, which is cumbersome, expensive and time-consuming. In contrast, the HPE has an advantage that it can be produced in relatively large quantities in a short amount of time, without the need for culturing. In addition, omitting the relatively harsh processes, such as hydrochloric acid hydrolysis and high-pressure steam sterilization, allows us to preserve more bioactive proteins, as shown in the present study. However, donors must be tested and confirmed to be negative for viral and bacterial infections by serological testing to eliminate the risk of infection.

Chemotherapy using anticancer drugs is a potentially life-extending treatment, while CIPN is one of the most common long-term side effects, including pain and numbness [37]. Although no effective strategies for preventing or treating CIPN have yet been developed, a large body of evidence suggests that oxidative damage is one of the critical factors involved in the neuropathic pain of CIPN patients [31] and that neuroprotection by increasing the neurite outgrowth contributes to alleviating mechanical allodynia [38]. Previous studies revealed that the placental extracts have antioxidant activity, and the main antioxidant components of these extracts are low-molecular-weight materials, such as uracil, tryptophan, tyrosine, phenylalanine, and collagen-derived peptides [39]. In addition, placental extracts were reported to delay in vitro cellular senescence through the activation of the nuclear factor erythroid 2-related factor 2-mediated antioxidant pathway [40]. Cow placental extract was reported to have protective effects on D-galactose-induced skin aging in mice through its strong free radical scavenging, reducing and metal chelating activities [41]. Moreover, to date, only a few papers have shown the neurite outgrowth activity of and alleviating activity [42] of HPE on neuropathic pain using mouse models, including peripheral nerve crush injury [43] and complete Freud’s adjuvant-induced inflammatory pain [44].

TRX-1 is a multifunctional 12 kDa protein with reduction-oxidation properties that is expressed throughout the cytosol and, in some cases, the nucleus [15,16,17]. Human TRX-1 consisting of 105 amino acids was initially identified as an adult T-cell leukemia-derived factor, ADF, in the supernatant of a T-cell line infected with HTLV-1. TRX-1 was found to be a secreted protein that stimulates IL-2 receptor α-chain expression, despite it not having a signaling peptide in its N-terminal. TRX-1 acts as a reducing factor and undergoes oxidation of the thiols (Cys32 and Cys35) present in the active site (Cys-Gly-Pro-Cys), forming a disulfide bond between them and leading to the loss of its reducing activity [16]. In general, the TRX-1 protein plays a critical role in a variety of cellular functions, including the maintenance of redox homeostasis, DNA synthesis, proliferation, the regulation of gene expression, and the control of cell death by apoptosis [16,17]. Several studies have suggested that TRX-1 may also have the ability to promote cell differentiation of neuronal cells. One such study, conducted on rat adrenal pheochromocytoma PC12 cells, showed that treating the cells with nerve growth factor resulted in increased TRX-1 expression and induced PC12 cell differentiation and neurite outgrowth [45]. Another study found that treating PC12 cells with TRX-1 induced cell differentiation and neurite outgrowth [46]. These reports suggest that TRX-1 might also contribute to alleviating activity of the HPE on mechanical allodynia.

Extracellular matrix proteins organize and regulate the extracellular microenvironment by interacting with cell surface receptors, growth factors, cytokines, and enzymes, thereby mediating communication between cells and their environment [47]. TSP-1 is a typical extracellular matrix protein that plays a role in regulating neural synapse formation, angiogenesis, platelet activation, wound healing, connective tissue organization, immunomodulation, and cell death [12]. TSP-1 interacts with multiple receptors, including CD47 and CD36, as well as with extracellular ligands, such as cytokines, growth factors, proteases and other cellular proteins, via its distinct domains [12,13,14]. The processing of proteins by proteases is an important post-translational mechanism that modulates the function of extracellular molecules, particularly those with multiple domains and active sites, such as TSP-1 [20,21]. Therefore, proteolytic fragmentation of extracellular matrix proteins has multiple functional consequences. Targeted and selective cleavage locally releases fragments or domains with enhanced, alternative or disrupted functions, thereby altering the composition and function of the their interacting microenvironment [48].

Although various HPEs have been prepared using different decellularization, extraction, and sterilization methods, most papers poorly describe the compositions of the resulting HPEs [8]. This is the first report on the proteomics analysis of HPE and the identification of its proteins. Previous papers have shown the results of proteomics analyses of HPE prepared using acid hydrolysis and autoclaving, therefore identifying only amino acids and some peptides [5,6]. Similar to this study, some of these previous reports have also shown the antioxidant and neurite outgrowth activities of HPE [5,22]. However, to the best of our knowledge, this is the first report on the therapeutic effects of HPE against hair graying and PTX-induced peripheral neuropathy using disease mouse models.

## 4. Materials and Methods

### 4.1. Cell Culture

Human neuroblastoma cell line SH-SY5Y (CRL-2266) [23,24], which was purchased from American Type Culture Collection (Manassas, VA, USA), was cultured in a 1:1 mixture of Dulbecco’s Modified Eagle Medium (DMEM, high glucose, Thermo Fisher Scientific, Waltham, MA, USA) and Ham’s F12 with L-glutamine (Fujifilm, Tokyo, Japan) (DMEM/F-12 medium), supplemented with fetal bovine serum (FBS, 10%) and penicillin (100 U/mL, Invitrogen, Carlsbad, CA, USA) and streptomycin (100 μg/mL, Invitrogen) at 37 °C in an atmosphere of 5% CO_2_/95% air.

### 4.2. Mice

C57BL/6J male mice, 6–7 weeks old, were purchased from Sankyo Labo Service (Hamamatsu, Japan). All mice were maintained under specific pathogen-free conditions, and all animal experiments were approved by the President and by the Institutional Animal Care and Use Committee of Tokyo Medical University (Approval numbers: Approval numbers: R5-100, R6-012, R7-015, R8-022 and R8-029) and were performed in accordance with institutional, science community, and national guidelines for animal experimentation and the Animal Research: Reporting of In Vivo Experiments guidelines 2.0.

### 4.3. Preparation of HPE

A new HPE called UPA^®^ was produced using human tissues from the placenta, umbilical cord and amnion. These tissues were donated by consenting individuals and were provided by Advanced Medical Tokyo Corporation and Japanese Society of Umbilical Cord and Placenta Medicine. This study was approved by the institutional review board of the Japan Council for Safety of Clinical Study (Approval numbers: 18000005). In brief, the cryopreserved tissues confirmed to be negative for viral and bacterial infections by serological testing were thawed, washed, minced, and homogenized, followed by hydrolysis with pepsin under acidic conditions. After centrifugation and neutralization, the hydrolyzate was incubated at 60 °C for 40 min, followed by filtration through 0.45 and 0.2 µm filters and lyophilization. Each lot was prepared from one donor. The lyophilized powder (with a net weight of approximately 0.28 g) was dissolved in 3 mL of PBS, with a small aliquot being frozen for later use. The protein contents of HPE lot #1 (11L221201) and #2 (13L041923) were determined to be 0.137 and 0.136 mg/mL, respectively, by the BCA Protein Assay Kit (Takara, Shiga, Japan) using bovine serum albumin as a standard protein. The HPE solutions as 100% were used after dilution with PBS or control medium (DMEM free of serum, phenol red, L-glutamine and sodium pyruvate, Thermo Fisher Scientific) in the present experiments.

### 4.4. SDS-PAGE and Western Blotting

The HPE samples were separated by SDS-PAGE under reducing conditions and subjected to protein staining with the Silver Stain MS Kit (Wako, Tokyo, Japan). For Western blotting, the separated proteins were transferred to a polyvinylidene difluoride membrane (Merck Millipore, Burlington, MA, USA), and the membrane was then blocked and probed with the following antibodies: goat polyclonal anti-TRX-1 (R&D Systems, Minneapolis, MN, USA) and goat polyclonal anti-TSP-1 (R&D Systems). Then, the membrane was incubated with anti-goat IgG antibody conjugated to horseradish peroxidase and visualized with an enhanced chemiluminescence detection system (GE Healthcare, Chicago, IL, USA) according to the manufacturer’s instructions. Immunoreactive bands were detected with an iBright FL1500 Imaging System (Thermo Fisher Scientific).

### 4.5. DIA Proteomic Analysis

DIA proteomic analysis was performed by Kazusa Genome Technologies, Inc. (Chiba, Japan) according to the previously reported method [17]. In brief, the HPE sample was reduced with tris(2-carboxyethyl)phosphine, followed by alkylation with iodoacetamide. Protein purification was performed using the sample preparation (SP3) method [49,50]. The tryptic digestion was performed using Trypsin/Lys-C Mix (Promega, Madison, WI, USA), followed by purification using GL-Tip SDB (GL Sciences, Tokyo, Japan). Subsequently, mass spectrometry measurements were performed by nano liquid chromatography with tandem MS (UltiMate 3000 RSLCnano LC system, Thermo Fisher Scientific). From the acquired MS data, peptides and proteins with both a peptide and protein false discovery rate below 1% were identified and quantified using Scaffold DIA (Proteome Software, Portland, OR, USA). The raw data were searched against an in silico predicted spectral library using DIA-NN (version: 1.8.1, https://github.com/vdemichev/DiaNN, downloaded on 26 November 2021) [51]. The in silico predicted spectral library was generated from the human protein sequence database (UniProt id UP000005640, reviewed, canonical, 20,381 entries) using DIA-NN. The protein identification threshold was set at <1% for both peptide and protein false discovery rates. Gene ontology analysis was performed by Metascape (http://metascape.org) [52].

### 4.6. ELISA

The contents of cytokines, hepatocyte growth factor (HGF), transforming growth factor (TGF)-β1, basic fibroblast growth factor (bFGF), interleukin (IL)-6, IGFBP-3, 4 and 6, tissue inhibitor of metalloproteinase (TIMP)-1, vascular endothelial growth factor (VEGF), TSP-1, PGRN, and TRX-1 were determined by respective sandwich ELISA kits according to the manufacturers’ instructions (R&D Systems, Biolegend, and Thermo Fisher Scientific).

### 4.7. Measurement of ROS Generation

The ROS levels generated by H_2_O_2_ in SH-SY5Y cells were analyzed using the 2′,7′-Dichlorofluorescein diacetate (DCFDA) Cellular ROS Detection Assay Kit (Abcam, Cambridge, UK), according to the manufacturer’s protocol. DCFDA is converted by ROS into the highly fluorescent DCF. After cells (6 × 10^4^) were seeded in complete DMEM/F-12 medium containing FBS (10%) in a 96-well plate (Greiner, Kremsmünster, Austria) and incubated overnight, the cells were preincubated with HPE (1%) or TRX-1 (1 µg/mL, R&D Systems) for 3 h. Thereafter, the cells were incubated with DCFDA (15 μM) solution for 30–45 min at 37 °C in the dark. After changing the DCFDA solution to HPE (1%) or TRX-1 (1 µg/mL), H_2_O_2_ (50 μM) was added. After 1 h, the cells were fixed and stained with Hoechst 33258 (Dojindo, Tokyo, Japan), and the fluorescence was measured using a microscope, BZ-X8100 (Keyence, Osaka, Japan). DCF and Hoechst 33258 signals were detected at Ex/Em = 470/525 nm and Ex/Em = 360/460 nm, respectively. Quantification of the fluorescence intensity was conducted with Fiji software (an expanded version of ImageJ, version 1.53c; National Institutes of Health, Bethesda, MD, USA).

### 4.8. Neurite Outgrowth Assay

Neurite outgrowth was determined as described previously [23,24], with slight modification as follows. In brief, SH-SY5Y cells were transfected with the GFP-expression plasmid DNA (pGFP, GFP cDNA subcloned in the pCAGGS vector) [53] using lipofectamine (Thermo Fisher Scientific), according to the manufacturer’s instructions, and plated (2 × 10^4^/well) in laminin- and poly-L-lysine-coated 24-well plates in DMEM/F-12 medium containing FBS (10%). After 1 day, the cells were stimulated with 10 µM ATRA (Sigma-Aldrich, St. Louis, MA, USA) in DMEM/F-12 medium containing FBS (2%). After 3 days, the medium was changed to DMEM/F-12 medium containing ATRA (10 µM) and FBS (1%) with and without HPE (5%) or BDNF (50 ng/mL, Biolegend, San Diego, CA, USA) as a positive control, PBS and cultured for an additional 3 days. Fluorescent images of the cells were acquired using a microscope EVOS M5000 Imaging System (Thermo Fisher Scientific). Neurite lengths of GFP^+^ neurite-bearing cells were measured by tracing and quantifying elongated image structures using NeuronJ, an ImageJ plugin (version 1.4.3; National Institutes of Health) [54].

### 4.9. Hair Graying Mouse Model Construction

The hair graying mouse model was developed through X-ray irradiation of C57BL/6J male mice after removal of dorsal hair, as reported previously [55]. Briefly, 6-week-old C57BL/6J mice were habituated for 1 week, and their dorsal hair was shaved at 7 weeks of age (day 0). Then, 100 µL of HPE (10%) or control medium was subcutaneously injected into the dorsal skin from day 1 every day for 1 week. The mice were randomly allocated to each group. On day 8, the dorsal hair was removed by waxing in order to induce the anagen hair cycle, and on day 9, the mice were irradiated with 5 Gy X-ray. During this period, 100 µL of HPE or control DMEM medium was subcutaneously injected into the dorsal skin every day from day 1 to day 19, and photographs of the dorsal area were taken with time. Hair blackness was assessed and quantified from each photograph with a CIELAB (CIE L*a*b*) color system [56] using Fiji software. The L* value indicates hair brightness from 0 (black) to 100 (white). The average L* value is shown as the result of the part of the dorsal area where the injection was performed.

### 4.10. CIPN Model Using PTX

The CIPN model was established as described previously [57]. Briefly, C57BL/6J male mice were intraperitoneally administered with PTX (8 mg/kg, AdipoGen, San Diego, CA, USA) dissolved in a solution of Cremophor EL (Selleck):ethanol:saline = 1:1:8 at a volume of 200 µL per mouse every other day for a total of 3~4 administrations. After around 10 days, the mice were administered intraperitoneally with 100 µL of HPE (10%) in PBS or PBS alone, with the number of administrations repeated as indicated. The mice were randomly allocated to each group.

### 4.11. Mechanical Allodynia Assay

Mechanical allodynia was assessed as a withdrawal response of the hind paw to the stimulation with von Frey filaments (Shin Factory, Fukuoka, Japan) using the up-and-down method as described previously [58,59]. A range of filaments from 0.02 g to 1.4 g was used, and the 0.6 g filament was the starting point. Fifty % paw withdrawal threshold was calculated from the response pattern using the Up-Down Reader software [59]. Although the mechanical allodynia was not assessed completely blindly, the objective scoring criteria were meticulously applied to minimize personal bias.

### 4.12. Immunohistochemical Analysis

The tissue samples from the skin on the plantar surface of the hind paws were harvested quickly and immediately embedded in the OCT compound, and the frozen samples were stored at −80 °C until further processing. For sectioning, 10 µm-thick sections were cut and mounted onto positively charged glass slides [60,61,62]. The sections were then air-dried for 60 min and stored at −80 °C until staining. For immunohistochemical analysis, the sections were fixed in neutral buffered formalin (10%) for 10 min and then washed with PBS. To retrieve the antigens, the sections were heated at 90 °C for 20 min in EDTA (0.5 M) pH 8.0 and then immediately cooled in cold PBS. The resulting sections were then permeabilized with Triton X-100 (0.2%) in PBS at room temperature for 30 min and blocked in PBS containing BSA (1%), Tween 20 (0.05%) and glycine (0.3 M) for 1 h. The sections were then incubated at 4 °C overnight with rabbit anti-protein gene product 9.5 (PGP9.5, Clone # EPR4118, Abcam) and rat anti-myelin basic protein (MBP, Clone #12, Abcam) in PBS containing BSA (1%) and Tween 20 (0.05%), followed by the appropriate secondary antibody. Images were captured using a microscope EVOS M5000 Imaging System (Thermo Fisher Scientific). The number of fibers crossing the dermo-epidermal junction was quantified. Specificially, each fiber that branched within the epidermal layer was counted as a single fiber, and fragments (>15 μm) were also included in the count. The lengths of the identified nerve fibers and fragments in the epidermis were measured using Fiji software. The number and total length of the fibers and fragments were normalized to the lenghth of the dermo-epidermal junction. Multiple sections per animal were analyzed.

### 4.13. Statistical Analysis

Data are described as the mean  ±  standard deviation of the mean (SD) for each group. Statistical analysis was performed with GraphPad Prism software (version 10; GraphPad Software, San Diego, CA, USA) using the unpaired two-tailed Student’s *t*-test for comparisons of two groups and one- or two-way analysis of variance with Tukey’s multiple comparisons test for comparisons involving more than three groups. *p* < 0.05 was considered statistically significant.

## 5. Conclusions

In the present study, we prepared a new HPE formulation under milder conditions, omitting acid hydrolysis and autoclaving, which appears to contain more intact and active high-molecular-weight proteins than those prepared previously. Consistent with this, the HPE induced both antioxidant and neurite outgrowth activities in an SH-SY5Y neuronal cell model. In addition, the HPE administration reduced hair graying caused by X-ray irradiation and alleviated CIPN with PTX in mice, possibly through its antioxidant and neurite outgrowth activities. Therefore, it has great potential to be used as a new therapeutic agent, although further studies on its therapeutic efficacy and safety are required.

## Data Availability

The original contributions presented in this study are included in the article and Supplementary Materials. Further inquiries can be directed to the corresponding author.

## Supplementary Materials

The following supporting information can be downloaded at: https://www.mdpi.com/article/10.3390/ijms27104188/s1.

## Abbreviations

The following abbreviations are used in this manuscript:

ATRA	all-trans-retinoic acid
bFGF	basic fibroblast growth factor
BDNF	brain-derived neurotrophic factor
CIPN	chemotherapy-induced peripheral neuropathy
DIA	data-independent acquisition
DCF	2′,7′-dichlorofluorescein
DCFDA	2′,7′-dichlorofluorescein diacetate
DMEM	Dulbecco’s Modified Eagle Medium
FBS	fetal bovine serum
HGF	hepatocyte growth factor
HPE	human placental extract
IGFBP	insulin-like growth factor binding protein
IL	interleukin
IENF	intraepidermal neuronal fiber
MBP	myelin basic protein
PBS	phosphate-buffered saline
PGRN	progranulin
PGP9.5	protein gene product 9.5
ROS	reactive oxygen species
SD	standard deviation
TIMP	tissue inhibitor of metalloproteinase
TRX-1	thioredoxin-1
TSP-1	thrombospondin-1
TGF	transforming growth factor
VEGF	vascular endothelial growth factor
