# Supplementary material for: Protective Effects of a New Human Placental Extract Against Hair Graying and Chemotherapy-Induced Peripheral Neuropathy
Source: Int J Mol Sci. 2026 May 8;27(10):4188. doi: 10.3390/ijms27104188 (PMC13206306; doi:10.3390/ijms27104188)
Supplement: Supplementary file 1 [file ijms-27-04188-s001.zip › ijms-4272139-supplementary.pdf]

Supplementary Material

Protective effects of a new human placental extract against hair graying and chemotherapy-induced peripheral neuropathy

Eri Horio[1], Yasuhiro Katahira[1], Natsuki Yamaguchi[1], Miki Igarashi[1], Hideaki Hasegawa[1], Satomi Miyakawa[1], Shota Toda[1], Izuru Mizoguchi[1], Ning Qu[1], Hiromitsu Anamizu[1], Shinichiro Ikeda[2], Hirohiko Matsumoto[3], and Takayuki Yoshimoto[1*]

[1]Department of Immunoregulation, Institute of Medical Science, Tokyo Medical University, 6-1-1 Shinjuku, Shinjuku-ku, Tokyo 160-8402, Japan

[2]Advanced Medical Tokyo Corporation, 2-6-1 Nihonbashi-Honcho, Chuo-ku, Tokyo 103-0023, Japan

[3]Japanese Society of Umbilical Cord and Placenta Medicine, 4-1 Kofuna-cho, Nihonbashi, Chuo-ku, Tokyo 103-0024, Japan

*Correspondence:
Takayuki Yoshimoto PhD, Department of Immunoregulation, Institute of Medical Science, Tokyo Medical University, 6-1-1 Shinjuku, Shinjuku-ku, Tokyo, Japan 160-8402, E-mail: yoshimot@tokyo-med.ac.jp

Supplementary Table S1. Comprehensive analyses of the protein composition of new HPE using data-independent acquisition (DIA) proteomics.

Ranking	Accession Number	Master Protein Name	Master Gene Symbol	MW (kDa)	Peptides	Unique Peptides	DIA Protein Intensity	Relative Intensity (%)
1	P02452	Collagen alpha-1(I) chain	COL1A1	138.942	15	13	3,992,270,000	15.93137633
2	P08123	Collagen alpha-2(I) chain	COL1A2	129.315	9	9	2,574,220,000	10.27256863
3	P02675	Fibrinogen beta chain	FGB	55.931	8	8	2,364,230,000	9.434591815
4	P02768	Albumin	ALB	69.365	30	30	2,200,280,000	8.780340186
5	P01834	Immunoglobulin kappa constant	IGKC	11.764	8	8	1,685,270,000	6.725164027
6	P02751	Fibronectin	FN1	272.323	44	44	1,475,050,000	5.88626938
7	P01009	Alpha-1-antitrypsin	SERPINA1	46.735	19	19	1,140,000,000	4.549233648
8	P01857	Immunoglobulin heavy constant gamma 1	IGHG1	36.105	11	6	898,000,000	3.583519137
9	P35555	Fibrillin-1	FBN1	312.307	50	49	609,349,000	2.431641206
10	P10599	Thioredoxin	TXN	11.737	3	3	538,049,000	2.147114575
11	P02787	Serotransferrin	TF	77.065	18	18	394,124,000	1.572773827
12	P02671	Fibrinogen alpha chain	FGA	94.975	12	12	375,432,000	1.498182357
13	P08572	Collagen alpha-2(IV) chain	COL4A2	167.555	5	5	301,930,000	1.204868522
14	P23142	Fibulin-1	FBLN1	77.216	9	9	281,448,000	1.123133958
15	P69892	Hemoglobin subunit gamma-2	HBG2	16.128	8	1	259,361,000	1.034994551
16	P18206	Vinculin	VCL	123.801	21	21	235,706,000	0.940597953
17	P24043	Laminin subunit alpha-2	LAMA2	343.911	37	37	220,148,000	0.878512885
18	P25815	Protein S100-P	S100P	10.4	4	4	212,216,000	0.846859796
19	P01859	Immunoglobulin heavy constant gamma 2	IGHG2	35.902	3	2	208,348,000	0.831424326
20	P09493	Tropomyosin alpha-1 chain	TPM1	32.708	7	5	178,171,000	0.711001323
21	P13727	Bone marrow proteoglycan	PRG2	25.205	6	6	169,542,000	0.676566817
22	B9A064;P0CG04	Immunoglobulin lambda-like polypeptide 5	IGLL5	23.061	4	1	152,521,000	0.608643566
23	P12111	Collagen alpha-3(VI) chain	COL6A3	343.671	34	34	146,935,000	0.586352321
24	P01860	Immunoglobulin heavy constant gamma 3	IGHG3	41.288	5	4	130,759,000	0.52180109
25	P35556	Fibrillin-2	FBN2	314.781	39	38	118,817,000	0.474145872
26	P11047	Laminin subunit gamma-1	LAMC1	177.608	22	22	105,470,000	0.420883924
27	P12110	Collagen alpha-2(VI) chain	COL6A2	108.582	6	6	100,320,000	0.400332561
28	A0A0A0MRZ8;P04433	Immunoglobulin kappa variable 3D-11	IGKV3D-11	12.624	1	1	99,682,200	0.397787385
29	P01008	Antithrombin-III	SERPINC1	52.604	12	12	93,337,800	0.372469702
30	P98160	Basement membrane-specific heparan sulfate proteoglycan core protein	HSPG2	468.836	32	32	83,329,700	0.33253182
31	P04004	Vitronectin	VTN	54.305	6	6	80,785,300	0.32237825
32	P67936	Tropomyosin alpha-4 chain	TPM4	28.519	4	2	78,105,500	0.311684359
33	P01011	Alpha-1-antichymotrypsin	SERPINA3	47.65	10	10	68,510,800	0.273396172
34	P61626	Lysozyme C	LYZ	16.537	2	2	65,512,500	0.261431289
35	Q14112	Nidogen-2	NID2	151.256	6	6	62,970,400	0.251286897
36	P02679	Fibrinogen gamma chain	FGG	51.511	6	6	60,276,900	0.240538335
37	P62805	Histone H4	H4-16	11.365	3	3	55,183,200	0.220211641
38	P08603	Complement factor H	CFH	139.097	22	18	52,716,600	0.210368536
39	P02461	Collagen alpha-1(III) chain	COL3A1	138.566	9	8	51,953,600	0.207323741
40	A0A075B6S2;A2NJV5	Immunoglobulin kappa variable 2D-29	IGKV2D-29	13.141	2	1	51,225,800	0.204419415
41	P08697	Alpha-2-antiplasmin	SERPINF2	54.565	4	4	48,822,900	0.194830508
42	P04264	Keratin, type II cytoskeletal 1	KRT1	66.038	16	15	47,919,200	0.191224243
43	Q06323	Proteasome activator complex subunit 1	PSME1	28.72	2	2	47,433,800	0.189287227
44	P35579	Myosin-9	MYH9	226.536	17	14	47,253,700	0.188568528

45	P07942	Laminin subunit beta-1	LAMB1	198.042	27	27	46,498,400	0.185554461
46	Q12805	EGF-containing fibulin-like extracellular matrix protein 1	EFEMP1	54.641	8	8	46,382,500	0.185091956
47	P00747	Plasminogen	PLG	90.569	17	17	45,889,200	0.183123415
48	Q9Y490	Talin-1	TLN1	269.773	11	10	45,492,100	0.181538765
49	P62753	40S ribosomal protein S6	RPS6	28.679	1	1	41,059,300	0.163849429
50	Q9Y4K1	Beta/gamma crystallin domain-containing protein 1	CRYBG1	188.679	1	1	39,815,400	0.158885577
51	P13987	CD59 glycoprotein	CD59	14.176	3	3	38,931,000	0.155356329
52	P13645	Keratin, type I cytoskeletal 10	KRT10	58.827	12	10	35,462,100	0.14151349
53	P35908	Keratin, type II cytoskeletal 2 epidermal	KRT2	65.433	20	14	35,325,500	0.14096838
54	A0A075B7D0	Immunoglobulin heavy variable 1/OR15-1 (non-functional) (Fragment)	IGHV1OR15-1	13.01	2	2	34,466,700	0.137541291
55	P55268	Laminin subunit beta-2	LAMB2	195.985	20	20	33,400,800	0.133287757
56	Q16363	Laminin subunit alpha-4	LAMA4	202.525	4	4	32,497,500	0.129683088
57	P01024	Complement C3	C3	187.152	22	22	31,042,700	0.123877628
58	P07093	Glia-derived nexin	SERPINE2	44.005	12	12	30,906,900	0.12333571
59	P30740	Leukocyte elastase inhibitor	SERPINB1	42.741	13	12	28,827,000	0.115035753
60	P02748	Complement component C9	C9	63.175	5	5	28,235,700	0.112676137
61	P52272	Heterogeneous nuclear ribonucleoprotein M	HNRNPM	77.518	1	1	26,790,200	0.106907789
62	Q13228	Methanethiol oxidase	SELENBP1	52.39	15	15	26,779,900	0.106866686
63	P80723	Brain acid soluble protein 1	BASP1	22.693	5	5	26,411,700	0.105397363
64	P0C0L4;P0C0L5	Complement C4-A	C4A	192.788	13	13	26,002,900	0.103766024
65	P35527	Keratin, type I cytoskeletal 9	KRT9	62.066	12	12	25,880,600	0.103277979
66	P02760	Protein AMBP	AMBP	39.001	4	4	25,716,100	0.102621533
67	P06312	Immunoglobulin kappa variable 4-1	IGKV4-1	13.379	2	2	25,372,600	0.101250777
68	P21333	Filamin-A	FLNA	280.742	25	24	24,009,800	0.095812447
69	P02538	Keratin, type II cytoskeletal 6A	KRT6A	60.045	14	2	23,568,300	0.094050617
70	P01876	Immunoglobulin heavy constant alpha 1	IGHA1	37.653	4	3	23,441,700	0.093545413
71	O15230	Laminin subunit alpha-5	LAMA5	399.744	32	32	22,606,400	0.090212101
72	P62263	40S ribosomal protein S14	RPS14	16.271	1	1	21,889,500	0.087351272
73	Q9Y6N6	Laminin subunit gamma-3	LAMC3	171.231	9	9	21,826,600	0.087100266
74	P01602	Immunoglobulin kappa variable 1-5	IGKV1-5	12.782	1	1	21,782,300	0.086923484
75	Q96P44	Collagen alpha-1(XXI) chain	COL21A1	99.37	2	2	21,761,700	0.086841279
76	Q14847	LIM and SH3 domain protein 1	LASP1	29.715	2	2	21,257,800	0.084830438
77	P68871	Hemoglobin subunit beta	HBB	15.997	4	1	21,189,300	0.084557085
78	P01772	Immunoglobulin heavy variable 3-33	IGHV3-33	13.073	3	1	21,105,600	0.084223075
79	P01023	Alpha-2-macroglobulin	A2M	163.292	31	27	21,020,700	0.083884277
80	Q15149	Plectin	PLEC	531.798	4	4	20,880,300	0.083324003
81	P13671	Complement component C6	C6	104.789	4	4	20,789,500	0.08296166
82	P10412	Histone H1.4	H1-4	21.864	3	1	20,566,600	0.082072166
83	P98095	Fibulin-2	FBLN2	126.574	9	9	19,854,200	0.079229294
84	P05155	Plasma protease C1 inhibitor	SERPING1	55.154	7	7	19,033,600	0.075954643
85	P21128	Uridylate-specific endoribonuclease	ENDOU	46.872	3	3	18,118,600	0.072303285
86	Q96Q89	Kinesin-like protein KIF20B	KIF20B	210.635	1	1	17,942,300	0.07159975
87	P03973	Antileukoproteinase	SLPI	14.326	3	3	17,494,400	0.06981238
88	P05556	Integrin beta-1	ITGB1	88.417	10	10	16,790,500	0.067003428

89	Q9BYB0	SH3 and multiple ankyrin repeat domains protein 3	SHANK3	184.669	1	1	16,372,100	0.065333779
90	P06753	Tropomyosin alpha-3 chain	TPM3	32.95	1	1	16,194,300	0.064624258
91	P20810	Calpastatin	CAST	76.573	5	5	15,911,300	0.063494931
92	P30101	Protein disulfide-isomerase A3	PDIA3	56.783	3	3	15,828,100	0.063162917
93	P61254	60S ribosomal protein L26	RPL26	17.259	2	1	15,457,700	0.061684815
94	P62854;Q5JNZ5	40S ribosomal protein S26	RPS26	13.012	1	1	15,269,700	0.06093459
95	A0A0C4DH25	Immunoglobulin kappa variable 3D-20	IGKV3D-20	12.513	1	1	14,864,500	0.059317617
96	P07858	Cathepsin B	CTSB	37.821	2	2	14,794,100	0.059036682
97	P17936	Insulin-like growth factor-binding protein 3	IGFBP3	31.673	5	5	14,763,300	0.058913773
98	P02533	Keratin, type I cytoskeletal 14	KRT14	51.559	15	5	14,631,300	0.05838702
99	A0A075B6R9;A0A0C4DH68	Probable non-functional immunoglobulin kappa variable 2D-24	IGKV2D-24	13.076	1	1	14,315,200	0.057125605
100	P23588	Eukaryotic translation initiation factor 4B	EIF4B	69.151	1	1	14,150,100	0.056466764
101	P13611	Versican core protein	VCAN	372.826	7	7	14,048,100	0.056059727
102	Q05682	Caldesmon	CALD1	93.231	5	5	13,689,200	0.054627517
103	P05023	Sodium/potassium-transporting ATPase subunit alpha-1	ATP1A1	112.897	1	1	13,433,000	0.053605136
104	P02647	Apolipoprotein A-I	APOA1	30.777	10	10	13,014,100	0.051933493
105	P18124	60S ribosomal protein L7	RPL7	29.225	4	4	12,825,600	0.051181273
106	P15529	Membrane cofactor protein	CD46	43.748	1	1	12,705,200	0.05070081
107	Q07954	Prolow-density lipoprotein receptor-related protein 1	LRP1	504.617	21	20	12,567,200	0.050150113
108	P05156	Complement factor I	CFI	65.75	6	6	12,242,500	0.04885438
109	Q14766	Latent-transforming growth factor beta-binding protein 1	LTBP1	186.797	14	14	12,225,200	0.048785343
110	P00441	Superoxide dismutase [Cu-Zn]	SOD1	15.935	2	2	12,099,300	0.048282932
111	P01780	Immunoglobulin heavy variable 3-7	IGHV3-7	12.941	2	1	12,044,600	0.048064649
112	Q6UY14	ADAMTS-like protein 4	ADAMTSL4	116.545	13	13	12,042,300	0.04805547
113	P80188	Neutrophil gelatinase-associated lipocalin	LCN2	22.589	7	7	11,962,000	0.047735029
114	Q8N2S1	Latent-transforming growth factor beta-binding protein 4	LTBP4	173.439	7	7	11,873,900	0.047383461
115	P16144	Integrin beta-4	ITGB4	202.17	7	7	11,706,300	0.046714644
116	P08174	Complement decay-accelerating factor	CD55	41.402	5	5	11,692,000	0.046657579
117	Q06830	Peroxiredoxin-1	PRDX1	22.111	4	3	11,589,100	0.046246951
118	Q5SSJ5	Heterochromatin protein 1-binding protein 3	HP1BP3	61.209	1	1	11,475,100	0.045792027
119	P02775	Platelet basic protein	PPBP	13.893	3	3	11,269,800	0.044972766
120	P00533	Epidermal growth factor receptor	EGFR	134.277	9	9	11,253,400	0.044907321
121	P29622	Kallistatin	SERPINA4	48.543	10	10	11,061,000	0.044139538
122	P43652	Afamin	AFM	69.072	5	5	10,516,200	0.041965483
123	P62841	40S ribosomal protein S15	RPS15	17.039	1	1	10,477,200	0.041809852
124	O75369	Filamin-B	FLNB	278.167	5	5	10,386,000	0.041445913
125	P22105	Tenascin-X	TNXB	458.395	14	13	10,210,700	0.040746368
126	P17900	Ganglioside GM2 activator	GM2A	20.839	3	3	10,117,900	0.040376045
127	Q03591	Complement factor H-related protein 1	CFHR1	37.653	1	1	10,058,700	0.040139804
128	Q14520	Hyaluronan-binding protein 2	HABP2	62.672	1	1	10,009,500	0.039943469
129	P61978	Heterogeneous nuclear ribonucleoprotein K	HNRNPK	50.979	2	2	9,987,930	0.039857392

130	Q05707	Collagen alpha-1(XIV) chain	COL14A1	193.519	9	9	9,724,510	0.0388062
131	Q6ZMP0	Thrombospondin type-1 domain-containing protein 4	THSD4	112.453	10	10	9,527,080	0.038018345
132	P50453	Serpin B9	SERPINB9	42.403	10	9	9,359,270	0.037348689
133	P25940	Collagen alpha-3(V) chain	COL5A3	172.122	1	1	9,236,270	0.036857851
134	P05154	Plasma serine protease inhibitor	SERPINA5	45.673	13	13	9,228,630	0.036827363
135	A0A0J9YX35	Immunoglobulin heavy variable 3-64D	IGHV3-64D	12.822	2	1	9,215,290	0.036774129
136	P13284	Gamma-interferon-inducible lysosomal thiol reductase	IFI30	27.964	1	1	9,076,610	0.036220719
137	P04275	von Willebrand factor	VWF	309.27	21	21	8,987,910	0.035866757
138	P32119	Peroxiredoxin-2	PRDX2	21.89	3	3	8,937,670	0.035666271
139	P00738	Haptoglobin	HP	45.205	7	3	8,922,810	0.035606971
140	Q14697	Neutral alpha-glucosidase AB	GANAB	106.874	4	4	8,711,540	0.034763887
141	Q9HC35	Echinoderm microtubule-associated protein-like 4	EML4	108.916	1	1	8,666,730	0.03458507
142	P01042	Kininogen-1	KNG1	71.959	6	6	8,264,290	0.032979111
143	P01700	Immunoglobulin lambda variable 1-47	IGLV1-47	12.283	2	1	8,186,910	0.032670321
144	P60709;P63261	Actin, cytoplasmic 1	ACTB	41.734	6	2	7,693,550	0.030701541
145	Q13151	Heterogeneous nuclear ribonucleoprotein A0	HNRNPA0	30.841	1	1	7,675,550	0.030629711
146	P51884	Lumican	LUM	38.43	5	5	7,571,390	0.030214055
147	Q9P2E9	Ribosome-binding protein 1	RRBP1	152.457	6	6	7,530,790	0.030052038
148	P10643	Complement component C7	C7	93.519	5	5	7,451,120	0.02973411
149	P05546	Heparin cofactor 2	SERPIND1	57.071	5	5	7,174,980	0.028632158
150	P07996	Thrombospondin-1	THBS1	129.385	15	15	7,082,190	0.028261875
151	Q16787	Laminin subunit alpha-3	LAMA3	366.659	5	5	6,979,480	0.027852005
152	P61916	NPC intracellular cholesterol transporter 2	NPC2	16.569	3	3	6,917,560	0.027604909
153	O00468	Agrin	AGRN	217.321	10	10	6,893,440	0.027508657
154	P35237	Serpin B6	SERPINB6	42.623	9	9	6,815,330	0.027196955
155	P00751	Complement factor B	CFB	85.534	2	2	6,790,090	0.027096233
156	Q14767	Latent-transforming growth factor beta-binding protein 2	LTBP2	195.055	6	6	6,776,140	0.027040565
157	P13473	Lysosome-associated membrane glycoprotein 2	LAMP2	44.962	3	3	6,754,470	0.02695409
158	O76021	Ribosomal L1 domain-containing protein 1	RSL1D1	54.974	1	1	6,715,720	0.026799456
159	A0A075B7B8	Immunoglobulin heavy variable 3/OR16-12 (non-functional) (Fragment)	IGHV3OR16-12	12.875	1	1	6,601,190	0.026342417
160	Q9BZP6	Acidic mammalian chitinase	CHIA	52.272	2	2	6,447,400	0.02572871
161	P04003	C4b-binding protein alpha chain	C4BPA	67.034	11	11	6,355,400	0.025361579
162	O43493	Trans-Golgi network integral membrane protein 2	TGOLN2	45.88	4	4	6,167,190	0.024610516
163	P08238	Heat shock protein HSP 90-beta	HSP90AB1	83.267	6	2	5,983,450	0.023877291
164	P45974	Ubiquitin carboxyl-terminal hydrolase 5	USP5	95.788	1	1	5,906,990	0.023572173
165	Q13219	Pappalysin-1	PAPPA	180.977	3	3	5,783,560	0.023079619
166	P13647	Keratin, type II cytoskeletal 5	KRT5	62.378	9	9	5,700,220	0.022747046
167	A0A087WSY6	Immunoglobulin kappa variable 3D-15	IGKV3D-15	12.533	1	1	5,681,930	0.022674059
168	O43148	mRNA cap guanine-N7 methyltransferase	RNMT	54.844	1	1	5,649,410	0.022544286
169	P29966	Myristoylated alanine-rich C-kinase substrate	MARCKS	31.554	3	3	5,647,540	0.022536824
170	P24821	Tenascin	TNC	240.857	10	10	5,555,090	0.022167897

171	P07358	Complement component C8 beta chain	C8B	67.045	3	3	5,525,700	0.022050614
172	A0A0B4J2B5	Immunoglobulin heavy variable 3/OR16-9 (non-functional) (Fragment)	IGHV3OR16-9	10.656	1	1	5,481,400	0.021873833
173	P48307	Tissue factor pathway inhibitor 2	TFPI2	26.933	1	1	5,424,670	0.021647449
174	P20908	Collagen alpha-1(V) chain	COL5A1	183.562	2	2	5,337,940	0.021301348
175	O95967	EGF-containing fibulin-like extracellular matrix protein 2	EFEMP2	49.404	2	2	5,325,270	0.021250787
176	P61353	60S ribosomal protein L27	RPL27	15.796	1	1	5,267,030	0.021018377
177	P27797	Calreticulin	CALR	48.141	1	1	5,259,900	0.020989925
178	Q13201	Multimerin-1	MMRN1	138.114	2	2	5,223,880	0.020846185
179	Q6ZRS2	Helicase SRCAP	SRCAP	343.561	1	1	5,180,710	0.020673913
180	A0A0A0MS15	Immunoglobulin heavy variable 3-49	IGHV3-49	13.056	4	3	5,125,810	0.020454831
181	Q6ZN17	Protein lin-28 homolog B	LIN28B	27.082	1	1	5,011,370	0.019998152
182	Q9UNF0	Protein kinase C and casein kinase substrate in neurons protein 2	PACSIN2	55.736	1	1	5,009,060	0.019988934
183	Q9Y624	Junctional adhesion molecule A	F11R	32.583	1	1	4,950,650	0.019755845
184	P05121	Plasminogen activator inhibitor 1	SERPINE1	45.06	6	6	4,937,500	0.019703369
185	P49908	Selenoprotein P	SELENOP	42.886	2	2	4,929,690	0.019672203
186	P05787	Keratin, type II cytoskeletal 8	KRT8	53.706	13	12	4,910,000	0.019593629
187	P25705	ATP synthase subunit alpha, mitochondrial	ATP5F1A	59.75	1	1	4,880,830	0.019477225
188	Q9H1E3	Nuclear ubiquitous casein and cyclin-dependent kinase substrate 1	NUCKS1	27.297	2	2	4,878,200	0.019466729
189	P35580	Myosin-10	MYH10	229.002	12	12	4,862,320	0.019403359
190	Q15404	Ras suppressor protein 1	RSU1	31.541	1	1	4,844,720	0.019333126
191	Q14624	Inter-alpha-trypsin inhibitor heavy chain H4	ITIH4	103.358	10	10	4,837,250	0.019303316
192	P16401	Histone H1.5	H1-5	22.579	1	1	4,773,090	0.019047282
193	Q13751	Laminin subunit beta-3	LAMB3	129.572	6	6	4,739,920	0.018914915
194	P05160	Coagulation factor XIII B chain	F13B	75.51	5	5	4,707,320	0.018784823
195	Q13443	Disintegrin and metalloproteinase domain-containing protein 9	ADAM9	90.559	2	2	4,669,990	0.018635856
196	P08729	Keratin, type II cytoskeletal 7	KRT7	51.385	11	9	4,618,370	0.018429863
197	P11279	Lysosome-associated membrane glycoprotein 1	LAMP1	44.882	3	3	4,586,700	0.018303482
198	P07910	Heterogeneous nuclear ribonucleoproteins C1/C2	HNRNPC	33.669	2	1	4,561,980	0.018204836
199	O94875	Sorbin and SH3 domain-containing protein 2	SORBS2	124.107	3	3	4,489,210	0.017914443
200	P27824	Calnexin	CANX	67.57	4	4	4,472,460	0.017847601
201	P36957	Dihydrolipoyllysine-residue succinyltransferase component of 2-oxoglutarate dehydrogenase complex, mitochondrial	DLST	48.758	3	3	4,458,270	0.017790975
202	P05120	Plasminogen activator inhibitor 2	SERPINB2	46.596	7	7	4,449,330	0.0177553
203	P02763	Alpha-1-acid glycoprotein 1	ORM1	23.512	5	4	4,392,080	0.01752684
204	Q13642	Four and a half LIM domains protein 1	FHL1	36.262	4	4	4,374,360	0.017456128
205	Q92522	Histone H1.10	H1-10	22.484	1	1	4,345,150	0.017339564
206	P13674	Prolyl 4-hydroxylase subunit alpha-1	P4HA1	61.052	1	1	4,313,530	0.017213382
207	P08727	Keratin, type I cytoskeletal 19	KRT19	44.106	4	3	4,293,600	0.017133851
208	Q68CZ2	Tensin-3	TNS3	155.268	1	1	4,242,820	0.01693121

209	Q9GZM7	Tubulointerstitial nephritis antigen-like	TINAGL1	52.387	4	4	4,234,700	0.016898807
210	P42766	60S ribosomal protein L35	RPL35	14.551	1	1	4,180,060	0.016680763
211	Q6H9L7	Isthmin-2	ISM2	63.908	1	1	4,177,910	0.016672183
212	Q99715	Collagen alpha-1(XII) chain	COL12A1	333.151	21	21	4,107,870	0.016392685
213	P02749	Beta-2-glycoprotein 1	APOH	38.3	3	3	4,084,080	0.016297749
214	P51608	Methyl-CpG-binding protein 2	MECP2	52.441	5	5	3,983,680	0.015897097
215	P02462	Collagen alpha-1(IV) chain	COL4A1	160.612	4	2	3,954,060	0.015778897
216	Q92896	Golgi apparatus protein 1	GLG1	134.556	4	4	3,849,010	0.015359689
217	Q9HBL0	Tensin-1	TNS1	185.704	1	1	3,848,210	0.015356497
218	P07951	Tropomyosin beta chain	TPM2	32.851	1	1	3,818,460	0.015237778
219	P11717	Cation-independent mannose-6-phosphate receptor	IGF2R	274.381	5	5	3,817,060	0.015232191
220	A0A0C4DH36	Probable non-functional immunoglobulin heavy variable 3-38	IGHV3-38	12.761	1	1	3,717,950	0.014836687
221	P00488	Coagulation factor XIII A chain	F13A1	83.268	3	3	3,703,400	0.014778624
222	Q9HD89	Resistin	RETN	11.419	2	2	3,655,950	0.014589273
223	P23246	Splicing factor, proline- and glutamine-rich	SFPQ	76.151	2	2	3,639,790	0.014524785
224	Q8TEA8	D-aminoacyl-tRNA deacylase 1	DTD1	23.424	2	2	3,630,610	0.014488152
225	P51858	Hepatoma-derived growth factor	HDGF	26.789	1	1	3,539,340	0.014123934
226	Q96HD1	Protein disulfide isomerase CRELD1	CRELD1	45.439	2	2	3,497,040	0.013955133
227	P01742	Immunoglobulin heavy variable 1-69	IGHV1-69	12.658	2	1	3,466,490	0.013833222
228	P42677	40S ribosomal protein S27	RPS27	9.46	1	1	3,454,550	0.013785575
229	P36980	Complement factor H-related protein 2	CFHR2	30.652	3	2	3,432,330	0.013696904
230	P01034	Cystatin-C	CST3	15.799	3	3	3,428,160	0.013680264
231	Q14204	Cytoplasmic dynein 1 heavy chain 1	DYNC1H1	532.418	3	3	3,414,340	0.013625114
232	Q86Y82	Syntaxin-12	STX12	31.64	3	3	3,367,120	0.01343668
233	P02730	Band 3 anion transport protein	SLC4A1	101.795	1	1	3,342,090	0.013336797
234	P02792	Ferritin light chain	FTL	20.018	2	2	3,333,680	0.013303236
235	O75882	Attractin	ATRN	158.542	7	7	3,300,220	0.013169712
236	P00734	Prothrombin	F2	70.037	3	3	3,244,640	0.012947917
237	Q01105	Protein SET	SET	33.487	2	2	3,218,170	0.012842287
238	Q4LDE5	Sushi, von Willebrand factor type A, EGF and pentraxin domain-containing protein 1	SVEP1	390.18	20	20	3,192,270	0.012738932
239	P00740	Coagulation factor IX	F9	51.78	4	4	3,169,830	0.012649384
240	P28799	Progranulin	GRN	63.544	5	5	3,155,890	0.012593755
241	Q92597	Protein NDRG1	NDRG1	42.836	1	1	3,118,770	0.012445626
242	Q9BXX0	EMILIN-2	EMILIN2	115.686	4	4	3,111,300	0.012415816
243	P56199	Integrin alpha-1	ITGA1	130.849	4	4	3,085,750	0.012313858
244	P55145	Mesencephalic astrocyte-derived neurotrophic factor	MANF	20.699	2	2	3,037,860	0.01212275
245	Q14315	Filamin-C	FLNC	291.025	15	15	3,022,730	0.012062373
246	P05090	Apolipoprotein D	APOD	21.276	4	4	3,016,880	0.012039028
247	P49747	Cartilage oligomeric matrix protein	COMP	82.864	5	5	2,993,710	0.011946567
248	A0A0C4DH38	Immunoglobulin heavy variable 5-51	IGHV5-51	12.673	2	1	2,993,240	0.011944691
249	P01782;P0DP04	Immunoglobulin heavy variable 3-9	IGHV3-9	12.945	1	1	2,976,020	0.011875974
250	Q9Y6R7	IgGFc-binding protein	FCGBP	572.028	8	8	2,963,970	0.011827888
251	P0DMV8;P0DMV9	Heat shock 70 kDa protein 1A	HSPA1A	70.056	2	1	2,956,770	0.011799156

252	P07355	Annexin A2	ANXA2	38.605	13	3	2,932,320	0.011701587
253	P14543	Nidogen-1	NID1	136.379	7	7	2,917,900	0.011644043
254	O95633	Follistatin-related protein 3	FSTL3	27.665	2	2	2,905,590	0.011594919
255	Q9BX66	Sorbin and SH3 domain-containing protein 1	SORBS1	142.514	4	4	2,887,270	0.011521812
256	P54652	Heat shock-related 70 kDa protein 2	HSPA2	70.02	2	2	2,851,570	0.011379349
257	P35749	Myosin-11	MYH11	227.342	8	8	2,757,370	0.011003439
258	P02790	Hemopexin	HPX	51.678	6	6	2,743,170	0.010946773
259	P15924	Desmoplakin	DSP	331.78	11	11	2,730,290	0.010895375
260	P02545	Prelamin-A/C	LMNA	74.141	8	8	2,726,300	0.010879452
261	A0A075B6H7	Probable non-functional immunoglobulin kappa variable 3-7	IGKV3-7	12.781	1	1	2,722,440	0.010864049
262	P43243	Matrin-3	MATR3	94.624	1	1	2,704,790	0.010793616
263	O76024	Wolframin	WFS1	100.293	1	1	2,681,450	0.010700476
264	Q13822	Ectonucleotide pyrophosphatase/phosphodiesterase family member 2	ENPP2	98.997	2	2	2,672,190	0.010663523
265	P26599	Polypyrimidine tract-binding protein 1	PTBP1	57.22	1	1	2,671,110	0.010659214
266	Q9NY15	Stabilin-1	STAB1	275.487	10	10	2,612,710	0.010426165
267	P00748	Coagulation factor XII	F12	67.792	1	1	2,592,150	0.010344119
268	Q9UPQ0	LIM and calponin homology domains-containing protein 1	LIMCH1	121.871	2	2	2,580,270	0.010296711
269	P04216	Thy-1 membrane glycoprotein	THY1	17.934	1	1	2,564,890	0.010235337
270	P08962	CD63 antigen	CD63	25.636	1	1	2,521,200	0.010060989
271	P68104;Q05639;Q5VTE0	Elongation factor 1-alpha 1	EEF1A1	50.139	2	2	2,501,220	0.009981258
272	Q8IUX7	Adipocyte enhancer-binding protein 1	AEBP1	130.931	1	1	2,483,750	0.009911543
273	P02774	Vitamin D-binding protein	GC	52.918	1	1	2,460,800	0.00981996
274	P21291	Cysteine and glycine-rich protein 1	CSRP1	20.567	4	4	2,455,280	0.009797932
275	P01019	Angiotensinogen	AGT	53.154	7	7	2,431,280	0.009702159
276	Q16270	Insulin-like growth factor-binding protein 7	IGFBP7	29.13	3	3	2,427,450	0.009686875
277	P62424	60S ribosomal protein L7a	RPL7A	29.994	2	2	2,414,430	0.009634918
278	P01714	Immunoglobulin lambda variable 3-19	IGLV3-19	12.041	1	1	2,389,760	0.009536471
279	P50452	Serpin B8	SERPINB8	42.767	4	4	2,351,930	0.009385508
280	P51991	Heterogeneous nuclear ribonucleoprotein A3	HNRNPA3	39.595	2	2	2,339,760	0.009336943
281	Q9C0C2	182 kDa tankyrase-1-binding protein	TNKS1BP1	181.799	3	3	2,331,550	0.00930418
282	Q02388	Collagen alpha-1(VII) chain	COL7A1	295.222	13	13	2,307,980	0.009210123
283	Q92945	Far upstream element-binding protein 2	KHSRP	73.117	1	1	2,306,930	0.009205933
284	Q14258	E3 ubiquitin/ISG15 ligase TRIM25	TRIM25	70.975	1	1	2,303,130	0.009190769
285	Q96GQ7	Probable ATP-dependent RNA helicase DDX27	DDX27	89.834	1	1	2,290,370	0.009139849
286	P47914	60S ribosomal protein L29	RPL29	17.751	1	1	2,278,070	0.009090766
287	Q96EI5	Transcription elongation factor A protein-like 4	TCEAL4	24.648	1	1	2,276,840	0.009085857
288	O75822	Eukaryotic translation initiation factor 3 subunit J	EIF3J	29.063	2	2	2,275,640	0.009081068
289	Q15124	Phosphoglucomutase-like protein 5	PGM5	62.225	12	12	2,264,140	0.009035177

290	P0DP23;P0DP24;P0DP25	Calmodulin-1	CALM1	16.836	1	1	2,257,050	0.009006884
291	P69905	Hemoglobin subunit alpha	HBA2	15.257	4	4	2,243,540	0.008952972
292	Q07666	KH domain-containing, RNA-binding, signal transduction-associated protein 1	KHDRBS1	48.225	2	2	2,217,300	0.008848259
293	O75339	Cartilage intermediate layer protein 1	CILP	132.566	2	2	2,204,770	0.008798258
294	Q53EL6	Programmed cell death protein 4	PDCD4	51.735	1	1	2,183,750	0.008714376
295	Q15256	Receptor-type tyrosine-protein phosphatase R	PTPRR	73.834	1	1	2,174,770	0.008678541
296	P48740	Mannan-binding lectin serine protease 1	MASP1	79.248	5	5	2,169,840	0.008658868
297	P11021	Endoplasmic reticulum chaperone BiP	HSPA5	72.331	4	3	2,169,480	0.008657431
298	Q1KMD3	Heterogeneous nuclear ribonucleoprotein U-like protein 2	HNRNPUL2	85.105	1	1	2,164,130	0.008636082
299	O43866	CD5 antigen-like	CD5L	38.085	1	1	2,158,890	0.008615171
300	Q04637	Eukaryotic translation initiation factor 4 gamma 1	EIF4G1	175.493	2	2	2,135,880	0.008523348
301	Q15746	Myosin light chain kinase, smooth muscle	MYLK	210.717	4	4	2,135,650	0.008522431
302	Q9NZV1	Cysteine-rich motor neuron 1 protein	CRIM1	113.74	2	2	2,134,840	0.008519198
303	P60981	Destrin	DSTN	18.506	2	2	2,120,610	0.008462413
304	P56181	NADH dehydrogenase [ubiquinone] flavoprotein 3, mitochondrial	NDUFV3	11.94	1	1	2,117,010	0.008448047
305	P04406	Glyceraldehyde-3-phosphate dehydrogenase	GAPDH	36.054	3	3	2,099,280	0.008377294
306	Q09666	Neuroblast differentiation-associated protein AHNAK	AHNAK	629.112	7	7	2,096,810	0.008367437
307	P01871	Immunoglobulin heavy constant mu	IGHM	49.44	5	5	2,095,460	0.00836205
308	Q15365	Poly(rC)-binding protein 1	PCBP1	37.497	3	3	2,093,470	0.008354109
309	Q07157	Tight junction protein ZO-1	TJP1	195.461	1	1	2,092,950	0.008352034
310	Q9UGM5	Fetuin-B	FETUB	42.055	1	1	2,092,350	0.008349639
311	O00515	Ladinin-1	LAD1	57.132	1	1	2,085,130	0.008320828
312	Q15113	Procollagen C-endopeptidase enhancer 1	PCOLCE	47.972	3	3	2,074,290	0.00827757
313	Q9Y2W1	Thyroid hormone receptor-associated protein 3	THRAP3	108.667	5	4	2,061,030	0.008224655
314	P98164	Low-density lipoprotein receptor-related protein 2	LRP2	521.97	6	6	2,058,230	0.008213482
315	P08047	Transcription factor Sp1	SP1	80.695	1	1	2,040,160	0.008141372
316	P14207	Folate receptor beta	FOLR2	29.276	3	3	2,034,510	0.008118826
317	Q02878	60S ribosomal protein L6	RPL6	32.728	2	2	2,011,890	0.008028559
318	P60468	Protein transport protein Sec61 subunit beta	SEC61B	9.974	1	1	2,005,420	0.00800274
319	Q9UM47	Neurogenic locus notch homolog protein 3	NOTCH3	243.637	5	5	1,995,710	0.007963992
320	Q14677	Clathrin interactor 1	CLINT1	68.26	3	3	1,976,330	0.007886655
321	P27918	Properdin	CFP	51.278	3	3	1,973,050	0.007873566
322	P35268	60S ribosomal protein L22	RPL22	14.786	1	1	1,943,930	0.007757361
323	P05543	Thyroxine-binding globulin	SERPINA7	46.325	5	5	1,935,060	0.007721965
324	Q96DZ1	Endoplasmic reticulum lectin 1	ERLEC1	54.859	1	1	1,931,470	0.007707639
325	Q12904	Aminoacyl tRNA synthase complex-interacting multifunctional protein 1	AIMP1	34.354	1	1	1,928,930	0.007697503

326	P06213	Insulin receptor	INSR	156.336	2	2	1,922,190	0.007670607
327	P02766	Transthyretin	TTR	15.885	2	2	1,913,340	0.00763529
328	P05455	Lupus La protein	SSB	46.835	1	1	1,909,910	0.007621602
329	Q13438	Protein OS-9	OS9	75.562	1	1	1,877,190	0.007491032
330	P18621	60S ribosomal protein L17	RPL17	21.397	2	2	1,875,540	0.007484447
331	Q07021	Complement component 1 Q subcomponent-binding protein, mitochondrial	C1QBP	31.362	2	2	1,853,150	0.007395099
332	Q16610	Extracellular matrix protein 1	ECM1	60.675	6	6	1,849,500	0.007380533
333	P24593	Insulin-like growth factor-binding protein 5	IGFBP5	30.569	1	1	1,820,060	0.007263051
334	O00567	Nucleolar protein 56	NOP56	66.051	1	1	1,805,310	0.00720419
335	P09661	U2 small nuclear ribonucleoprotein A'	SNRPA1	28.415	1	1	1,799,100	0.007179409
336	P04792	Heat shock protein beta-1	HSPB1	22.783	1	1	1,795,400	0.007164644
337	Q9BU23	Lipase maturation factor 2	LMF2	79.698	1	1	1,794,370	0.007160534
338	P05187;P10696	Alkaline phosphatase, placental type	ALPP	57.955	2	1	1,788,590	0.007137468
339	P05067	Amyloid-beta precursor protein	APP	86.944	3	3	1,769,170	0.007059972
340	P08833	Insulin-like growth factor-binding protein 1	IGFBP1	27.904	1	1	1,767,090	0.007051671
341	P02788	Lactotransferrin	LTF	78.183	5	5	1,762,570	0.007033634
342	P62249	40S ribosomal protein S16	RPS16	16.444	1	1	1,741,340	0.006948914
343	P10586	Receptor-type tyrosine-protein phosphatase F	PTPRF	212.882	2	2	1,733,140	0.006916192
344	Q01844	RNA-binding protein EWS	EWSR1	68.481	1	1	1,720,950	0.006867547
345	P35221	Catenin alpha-1	CTNNA1	100.073	3	3	1,717,220	0.006852662
346	Q15075	Early endosome antigen 1	EEA1	162.468	12	12	1,709,090	0.006820219
347	P02765	Alpha-2-HS-glycoprotein	AHSG	39.342	3	3	1,703,430	0.006797633
348	O43143	Pre-mRNA-splicing factor ATP-dependent RNA helicase DHX15	DHX15	90.934	1	1	1,700,730	0.006786858
349	Q86WC4	Osteopetrosis-associated transmembrane protein 1	OSTM1	37.256	1	1	1,683,250	0.006717103
350	O60504	Vinexin	SORBS3	75.341	1	1	1,675,440	0.006685937
351	Q9BTC0	Death-inducer obliterator 1	DIDO1	243.876	1	1	1,664,360	0.006641722
352	P82979	SAP domain-containing ribonucleoprotein	SARNP	23.672	1	1	1,658,670	0.006619015
353	O95400	CD2 antigen cytoplasmic tail-binding protein 2	CD2BP2	37.647	1	1	1,641,030	0.006548622
354	P13497	Bone morphogenetic protein 1	BMP1	111.25	2	2	1,630,030	0.006504726
355	Q9H8L6	Multimerin-2	MMRN2	104.41	2	2	1,623,890	0.006480224
356	P08069	Insulin-like growth factor 1 receptor	IGF1R	154.798	1	1	1,618,850	0.006460111
357	A0A0B4J1X8	Immunoglobulin heavy variable 3-43	IGHV3-43	13.077	1	1	1,617,220	0.006453607
358	O43291	Kunitz-type protease inhibitor 2	SPINT2	28.229	1	1	1,593,710	0.006359789
359	O75533	Splicing factor 3B subunit 1	SF3B1	145.831	2	2	1,591,920	0.006352646
360	Q13404	Ubiquitin-conjugating enzyme E2 variant 1	UBE2V1	16.495	2	1	1,591,730	0.006351887
361	Q93091	Ribonuclease K6	RNASE6	17.197	2	2	1,582,300	0.006314256
362	P08185	Corticosteroid-binding globulin	SERPINA6	45.14	3	3	1,582,110	0.006313498
363	P31949	Protein S100-A11	S100A11	11.742	2	2	1,549,970	0.006185242
364	O14672	Disintegrin and metalloproteinase domain-containing protein 10	ADAM10	84.14	3	3	1,546,680	0.006172113
365	Q2M2I5	Keratin, type I cytoskeletal 24	KRT24	55.086	1	1	1,533,210	0.00611836
366	Q9H4G0	Band 4.1-like protein 1	EPB41L1	98.504	1	1	1,512,980	0.006037631
367	P09497	Clathrin light chain B	CLTB	25.19	1	1	1,503,430	0.005999521

368	Q9NX58	Cell growth-regulating nucleolar protein	LYAR	43.616	1	1	1,499,280	0.005982961
369	P00742	Coagulation factor X	F10	54.731	2	2	1,494,440	0.005963646
370	P78527	DNA-dependent protein kinase catalytic subunit	PRKDC	469.099	1	1	1,481,440	0.005911769
371	Q9Y2W2	WW domain-binding protein 11	WBP11	69.997	2	2	1,479,010	0.005902072
372	A0A0B4J1U7	Immunoglobulin heavy variable 6-1	IGHV6-1	13.48	1	1	1,468,320	0.005859413
373	O95817	BAG family molecular chaperone regulator 3	BAG3	61.595	2	2	1,463,070	0.005838463
374	Q96HY6	DDRGK domain-containing protein 1	DDRGK1	35.61	1	1	1,446,930	0.005774055
375	P12724	Eosinophil cationic protein	RNASE3	18.384	2	2	1,445,320	0.00576763
376	P16284	Platelet endothelial cell adhesion molecule	PECAM1	82.524	2	2	1,443,430	0.005760088
377	Q9NZ45	CDGSH iron-sulfur domain-containing protein 1	CISD1	12.199	1	1	1,438,420	0.005740095
378	P78539	Sushi repeat-containing protein SRPX	SRPX	51.573	4	4	1,433,470	0.005720342
379	P10636	Microtubule-associated protein tau	MAPT	78.928	3	3	1,419,150	0.005663197
380	P19652	Alpha-1-acid glycoprotein 2	ORM2	23.603	3	3	1,415,410	0.005648273
381	P16109	P-selectin	SELP	90.835	3	3	1,404,400	0.005604337
382	Q00888	Pregnancy-specific beta-1-glycoprotein 4	PSG4	47.113	1	1	1,400,620	0.005589252
383	A0A0B4J1V0	Immunoglobulin heavy variable 3-15	IGHV3-15	12.926	2	2	1,391,200	0.005551661
384	P08514	Integrin alpha-IIb	ITGA2B	113.378	1	1	1,386,970	0.005534781
385	Q14126	Desmoglein-2	DSG2	122.295	2	2	1,382,160	0.005515587
386	Q3YEC7	Rab-like protein 6	RABL6	79.549	1	1	1,361,200	0.005431945
387	Q2M1K9;Q96K83	Zinc finger protein 423	ZNF423	144.607	1	1	1,360,210	0.005427994
388	Q86SQ4	Adhesion G-protein coupled receptor G6	ADGRG6	136.698	2	2	1,359,280	0.005424283
389	Q9Y3B3	Transmembrane emp24 domain-containing protein 7	TMED7	25.171	3	3	1,356,070	0.005411473
390	P01703	Immunoglobulin lambda variable 1-40	IGLV1-40	12.301	1	1	1,353,640	0.005401776
391	P42166	Lamina-associated polypeptide 2, isoform alpha	TMPO	75.492	2	2	1,353,220	0.0054001
392	Q9Y2V2	Calcium-regulated heat-stable protein 1	CARHSP1	15.891	1	1	1,332,740	0.005318373
393	Q04721	Neurogenic locus notch homolog protein 2	NOTCH2	265.41	5	3	1,331,770	0.005314503
394	Q9NPF0	CD320 antigen	CD320	28.99	1	1	1,331,380	0.005312946
395	P07225	Vitamin K-dependent protein S	PROS1	75.123	2	2	1,326,890	0.005295029
396	A0A0A0MT36;A0A0C4DH26	Immunoglobulin kappa variable 6D-21	IGKV6D-21	12.339	1	1	1,325,480	0.005289402
397	P05783	Keratin, type I cytoskeletal 18	KRT18	48.056	3	2	1,325,020	0.005287566
398	Q12841	Follistatin-related protein 1	FSTL1	34.989	3	3	1,311,910	0.00523525
399	P08779	Keratin, type I cytoskeletal 16	KRT16	51.266	5	4	1,310,560	0.005229863
400	Q01459	Di-N-acetylchitobiase	CTBS	43.76	4	4	1,299,910	0.005187363
401	P07360	Complement component C8 gamma chain	C8G	22.276	5	5	1,296,520	0.005173835
402	Q9BPY8	Homeodomain-only protein	HOPX	8.256	1	1	1,292,320	0.005157075
403	Q02447	Transcription factor Sp3	SP3	81.927	1	1	1,271,420	0.005073672
404	O75223	Gamma-glutamylcyclotransferase	GGCT	21.007	4	4	1,267,470	0.00505791
405	P30041	Peroxiredoxin-6	PRDX6	25.034	2	2	1,266,470	0.005053919
406	Q86XP3	ATP-dependent RNA helicase DDX42	DDX42	102.978	1	1	1,264,440	0.005045818

407	O60829	P antigen family member 4	PAGE4	11.151	2	2	1,258,750	0.005023112
408	Q8WW12	PEST proteolytic signal-containing nuclear protein	PCNP	18.926	2	2	1,253,340	0.005001523
409	P08138	Tumor necrosis factor receptor superfamily member 16	NGFR	45.184	1	1	1,246,010	0.004972272
410	P20160	Azurocidin	AZU1	26.884	1	1	1,245,380	0.004969758
411	O14684	Prostaglandin E synthase	PTGES	17.101	1	1	1,234,230	0.004925264
412	O00391	Sulfhydryl oxidase 1	QSOX1	82.576	3	3	1,229,750	0.004907386
413	P50479	PDZ and LIM domain protein 4	PDLIM4	35.396	1	1	1,223,430	0.004882166
414	Q9UM22	Mammalian ependymin-related protein 1	EPDR1	25.436	4	4	1,222,250	0.004877457
415	P50395	Rab GDP dissociation inhibitor beta	GDI2	50.666	1	1	1,218,440	0.004862253
416	Q13428	Treacle protein	TCOF1	152.11	2	2	1,208,080	0.004820911
417	P53634	Dipeptidyl peptidase 1	CTSC	51.853	2	2	1,204,080	0.004804948
418	Q96IZ0	PRKC apoptosis WT1 regulator protein	PAWR	36.566	2	2	1,203,240	0.004801596
419	Q15063	Periostin	POSTN	93.317	1	1	1,198,090	0.004781045
420	Q13361	Microfibrillar-associated protein 5	MFAP5	19.608	1	1	1,196,700	0.004775498
421	P10909	Clusterin	CLU	52.494	3	3	1,189,640	0.004747325
422	P08842	Steryl-sulfatase	STS	65.493	2	2	1,162,950	0.004640817
423	Q8NE71	ATP-binding cassette sub-family F member 1	ABCF1	95.927	1	1	1,156,850	0.004616475
424	Q9H0B8	Cysteine-rich secretory protein LCCL domain-containing 2	CRISPLD2	55.921	1	1	1,147,480	0.004579083
425	Q14515	SPARC-like protein 1	SPARCL1	75.208	3	3	1,145,490	0.004571142
426	P23229	Integrin alpha-6	ITGA6	126.604	1	1	1,134,130	0.004525809
427	Q9NZT2	Opioid growth factor receptor	OGFR	73.326	1	1	1,133,940	0.004525051
428	P05106	Integrin beta-3	ITGB3	87.058	2	2	1,133,780	0.004524412
429	P18887	DNA repair protein XRCC1	XRCC1	69.475	2	2	1,131,090	0.004513678
430	Q9NS15	Latent-transforming growth factor beta-binding protein 3	LTBP3	139.36	4	4	1,117,470	0.004459326
431	O95359	Transforming acidic coiled-coil-containing protein 2	TACC2	309.432	3	3	1,113,360	0.004442925
432	P36578	60S ribosomal protein L4	RPL4	47.696	1	1	1,112,240	0.004438456
433	P22897	Macrophage mannose receptor 1	MRC1	166.013	1	1	1,101,820	0.004396874
434	P10153	Non-secretory ribonuclease	RNASE2	18.354	2	2	1,097,920	0.004381311
435	P05164	Myeloperoxidase	MPO	83.872	5	4	1,095,360	0.004371095
436	P37837	Transaldolase	TALDO1	37.54	8	8	1,095,100	0.004370058
437	P06681	Complement C2	C2	83.269	1	1	1,092,960	0.004361518
438	P14625	Endoplasmin	HSP90B1	92.472	2	2	1,090,960	0.004353537
439	P12109	Collagen alpha-1(VI) chain	COL6A1	108.529	3	3	1,086,000	0.004333744
440	Q92820	Gamma-glutamyl hydrolase	GGH	35.965	5	5	1,083,970	0.004325643
441	Q92743	Serine protease HTRA1	HTRA1	51.287	1	1	1,079,140	0.004306368
442	Q9P273	Teneurin-3	TENM3	300.955	2	2	1,078,060	0.004302059
443	P51825	AF4/FMR2 family member 1	AFF1	131.422	1	1	1,068,860	0.004265346
444	O43237	Cytoplasmic dynein 1 light intermediate chain 2	DYNC1LI2	54.101	2	2	1,067,940	0.004261674
445	Q15366	Poly(rC)-binding protein 2	PCBP2	38.579	2	2	1,064,250	0.004246949
446	Q4VCS5;Q8IY63	Angiomotin	AMOT	118.085	1	1	1,048,010	0.004182142
447	P06731;P13688;P31997	Carcinoembryonic antigen-related cell adhesion molecule 5	CEACAM5	76.794	1	1	1,033,690	0.004124998
448	Q9NUM4	Transmembrane protein 106B	TMEM106B	31.13	1	1	1,020,440	0.004072123
449	P62820	Ras-related protein Rab-1A	RAB1A	22.675	1	1	1,006,040	0.004014659
450	O15347	High mobility group protein B3	HMGB3	22.979	1	1	1,005,960	0.00401434
451	Q00341	Vigilin	HDLBP	141.457	1	1	993,239	0.003963576

452	P07900;Q58FG0	Heat shock protein HSP 90-alpha	HSP90AA1	84.662	2	2	984,883	0.003930231
453	P02795;P04732;P13640;P80297;Q8N339	Metallothionein-2	MT2A	6.04	1	1	983,902	0.003926316
454	Q9UHB6	LIM domain and actin-binding protein 1	LIMA1	85.228	3	3	963,950	0.003846696
455	O43491	Band 4.1-like protein 2	EPB41L2	112.588	1	1	960,656	0.003833551
456	P07585	Decorin	DCN	39.745	2	2	957,105	0.003819381
457	P60174	Triosephosphate isomerase	TPI1	26.669	2	2	956,720	0.003817845
458	P78417	Glutathione S-transferase omega-1	GSTO1	27.565	4	4	947,000	0.003779056
459	P18031	Tyrosine-protein phosphatase non-receptor type 1	PTPN1	49.968	2	2	939,182	0.003747858
460	P11171	Protein 4.1	EPB41	97.018	1	1	938,331	0.003744462
461	Q9BQ69	ADP-ribose glycohydrolase MACROD1	MACROD1	35.505	1	1	936,914	0.003738808
462	Q9UBX5	Fibulin-5	FBLN5	50.181	1	1	935,309	0.003732403
463	Q6PJT7	Zinc finger CCCH domain-containing protein 14	ZC3H14	82.878	1	1	933,468	0.003725056
464	Q9H444	Charged multivesicular body protein 4b	CHMP4B	24.951	1	1	932,756	0.003722215
465	Q9H3G5	Probable serine carboxypeptidase CPVL	CPVL	54.163	2	2	932,654	0.003721808
466	Q53SF7	Cordon-bleu protein-like 1	COBLL1	123.869	2	2	928,721	0.003706113
467	O60814;P06899;P23527;P33778;P57053;P58876;P62807;Q16778;Q5QNW6;Q8N257;Q93079;Q96A08;Q99877;Q99879;Q99880	Histone H2B type 1-K	H2BC12	13.889	1	1	921,790	0.003678454
468	O75563	Src kinase-associated phosphoprotein 2	SKAP2	41.218	2	2	921,464	0.003677154
469	Q9NZN5	Rho guanine nucleotide exchange factor 12	ARHGEF12	173.237	1	1	920,111	0.003671754
470	Q9Y5L4	Mitochondrial import inner membrane translocase subunit Tim13	TIMM13	10.502	1	1	919,509	0.003669352
471	P48729	Casein kinase I isoform alpha	CSNK1A1	38.915	1	1	918,599	0.003665721
472	Q96TA1	Protein Niban 2	NIBAN2	84.137	1	1	910,247	0.003632391
473	Q7L2J0	7SK snRNA methylphosphate capping enzyme	MEPCE	74.356	1	1	909,061	0.003627659
474	O95274	Ly6/PLAUR domain-containing protein 3	LYPD3	35.968	1	1	898,999	0.003587506
475	P54819	Adenylate kinase 2, mitochondrial	AK2	26.478	1	1	894,415	0.003569213
476	P17096	High mobility group protein HMG-I/HMG-Y	HMGA1	11.675	1	1	882,532	0.003521793
477	Q9BXI6	TBC1 domain family member 10A	TBC1D10A	57.118	1	1	879,019	0.003507774
478	P00915	Carbonic anhydrase 1	CA1	28.87	2	2	877,803	0.003502922
479	Q9NPY3	Complement component C1q receptor	CD93	68.56	1	1	875,774	0.003494825
480	Q9Y520	Protein PRRC2C	PRRC2C	316.918	2	2	872,199	0.003480559
481	O43920	NADH dehydrogenase [ubiquinone] iron-sulfur protein 5	NDUFS5	12.517	1	1	870,164	0.003472438
482	Q8IUR6	CREB3 regulatory factor	CREBRF	72.147	1	1	869,023	0.003467885
483	Q8NHP8	Putative phospholipase B-like 2	PLBD2	65.469	4	4	864,103	0.003448251
484	P12270	Nucleoprotein TPR	TPR	267.298	3	3	863,098	0.003444241
485	O75629	Protein CREG1	CREG1	24.074	3	3	858,471	0.003425776
486	P25311	Zinc-alpha-2-glycoprotein	AZGP1	34.26	4	4	849,666	0.00339064

487	A0A075B6K4	Immunoglobulin lambda variable 3-10	IGLV3-10	12.438	2	1	839,909	0.003351704
488	Q8WWI1	LIM domain only protein 7	LMO7	192.702	1	1	836,944	0.003339872
489	Q52LJ0;Q8NCA5	Protein FAM98B	FAM98B	45.546	1	1	834,940	0.003331875
490	P69891	Hemoglobin subunit gamma-1	HBG1	16.142	1	1	832,744	0.003323111
491	Q14993	Collagen alpha-1(XIX) chain	COL19A1	115.221	1	1	831,324	0.003317445
492	P62937	Peptidyl-prolyl cis-trans isomerase A	PPIA	18.013	2	2	827,583	0.003302516
493	P35611	Alpha-adducin	ADD1	80.956	2	2	824,493	0.003290185
494	P29279	CCN family member 2	CCN2	38.091	3	3	823,021	0.003284311
495	Q14789	Golgin subfamily B member 1	GOLGB1	376.027	2	2	821,606	0.003278665
496	Q14008	Cytoskeleton-associated protein 5	CKAP5	225.499	1	1	820,999	0.003276242
497	Q06124	Tyrosine-protein phosphatase non-receptor type 11	PTPN11	68.012	1	1	817,579	0.003262595
498	P68431;P84243;Q16695;Q5TEC6;Q6NXT2;Q71DI3	Histone H3.1	H3C12	15.402	1	1	814,400	0.003249909
499	Q14980	Nuclear mitotic apparatus protein 1	NUMA1	238.263	4	4	812,133	0.003240862
500	P17844	Probable ATP-dependent RNA helicase DDX5	DDX5	69.148	2	1	810,731	0.003235267
501	P00450	Ceruloplasmin	CP	122.207	9	9	808,627	0.003226871
502	Q9P0M6	Core histone macro-H2A.2	MACROH2A2	40.056	1	1	805,285	0.003213535
503	Q04756	Hepatocyte growth factor activator	HGFAC	70.68	1	1	800,903	0.003196048
504	Q9BXP8	Pappalysin-2	PAPPA2	198.54	2	2	799,433	0.003190182
505	P36871	Phosphoglucomutase-1	PGM1	61.45	1	1	797,619	0.003182943
506	P07357	Complement component C8 alpha chain	C8A	65.165	4	4	792,351	0.003161921
507	Q9UHD8	Septin-9	SEPTIN9	65.402	1	1	792,021	0.003160604
508	Q9NWH9	SAFB-like transcription modulator	SLTM	117.151	1	1	790,698	0.003155325
509	Q15582	Transforming growth factor-beta-induced protein ig-h3	TGFBI	74.68	1	1	786,987	0.003140516
510	Q9UNK0	Syntaxin-8	STX8	26.905	1	1	783,086	0.003124948
511	Q15642	Cdc42-interacting protein 4	TRIP10	68.351	1	1	783,024	0.003124701
512	Q12913	Receptor-type tyrosine-protein phosphatase eta	PTPRJ	145.944	3	3	781,041	0.003116788
513	P06396	Gelsolin	GSN	85.699	1	1	779,360	0.00311008
514	Q13867	Bleomycin hydrolase	BLMH	52.563	1	1	778,616	0.003107111
515	O00339	Matrilin-2	MATN2	106.839	2	2	768,679	0.003067456
516	Q8NBJ4	Golgi membrane protein 1	GOLM1	45.332	1	1	762,266	0.003041865
517	P25398	40S ribosomal protein S12	RPS12	14.513	2	2	762,169	0.003041478
518	Q9C0J8	pre-mRNA 3' end processing protein WDR33	WDR33	145.891	1	1	761,646	0.003039391
519	P04040	Catalase	CAT	59.757	5	5	758,215	0.003025699
520	P04083	Annexin A1	ANXA1	38.714	4	4	755,071	0.003013153
521	Q16658	Fascin	FSCN1	54.531	1	1	755,002	0.003012878
522	P08670	Vimentin	VIM	53.652	6	4	750,209	0.002993751
523	O00425	Insulin-like growth factor 2 mRNA-binding protein 3	IGF2BP3	63.705	2	2	747,601	0.002983344
524	A0A0C4DH72;A0A0C4DH73;P01597;P01611;P04432	Immunoglobulin kappa variable 1-6	IGKV1-6	12.698	2	2	743,784	0.002968112
525	P15848	Arylsulfatase B	ARSB	59.688	4	4	733,494	0.002927049
526	Q9UPN3	Microtubule-actin cross-linking factor 1, isoforms 1/2/3/5	MACF1	838.323	2	2	733,224	0.002925971
527	Q13492	Phosphatidylinositol-binding clathrin assembly protein	PICALM	70.755	1	1	727,152	0.002901741

528	Q01085	Nucleolysin TIAR	TIAL1	41.589	1	1	726,395	0.00289872
529	P57772	Selenocysteine-specific elongation factor	EEFSEC	65.306	1	1	718,177	0.002865925
530	Q9UGM3	Deleted in malignant brain tumors 1 protein	DMBT1	260.74	1	1	714,527	0.00285136
531	P04114	Apolipoprotein B-100	APOB	515.615	17	17	709,260	0.002830342
532	Q9BXR6	Complement factor H-related protein 5	CFHR5	64.42	3	3	693,539	0.002767606
533	Q9NYF8	Bcl-2-associated transcription factor 1	BCLAF1	106.124	2	2	687,428	0.00274322
534	Q9Y282	Endoplasmic reticulum-Golgi intermediate compartment protein 3	ERGIC3	43.221	1	1	681,976	0.002721463
535	P99999	Cytochrome c	CYCS	11.746	2	2	678,534	0.002707728
536	Q00577	Transcriptional activator protein Pur-alpha	PURA	34.908	1	1	676,889	0.002701163
537	P51888	Prolargin	PRELP	43.812	1	1	675,403	0.002695233
538	A0A0B4J1Y9	Immunoglobulin heavy variable 3-72	IGHV3-72	13.202	1	1	656,330	0.002619122
539	Q96HE7	ERO1-like protein alpha	ERO1A	54.392	1	1	655,615	0.002616268
540	Q13409	Cytoplasmic dynein 1 intermediate chain 2	DYNC1I2	71.459	1	1	646,857	0.002581319
541	P35612	Beta-adducin	ADD2	80.856	1	1	641,527	0.002560049
542	Q8TDM6	Disks large homolog 5	DLG5	213.869	2	2	629,707	0.002512881
543	B5ME19;Q99613	Eukaryotic translation initiation factor 3 subunit C-like protein	EIF3CL	105.475	1	1	628,655	0.002508683
544	Q641Q2	WASH complex subunit 2A	WASHC2A	147.186	3	1	624,024	0.002490203
545	Q7KZI7	Serine/threonine-protein kinase MARK2	MARK2	87.911	2	2	623,328	0.002487425
546	Q5H9L2;Q6IPX3;Q969E4	Transcription elongation factor A protein-like 5	TCEAL5	23.306	1	1	613,466	0.00244807
547	A0A0J9YY99	Ig-like domain-containing protein (Fragment)	zzzz	12.962	1	1	603,472	0.002408189
548	Q9H013	Disintegrin and metalloproteinase domain-containing protein 19	ADAM19	104.999	1	1	600,450	0.002396129
549	P19634	Sodium/hydrogen exchanger 1	SLC9A1	90.765	1	1	598,121	0.002386835
550	P35573	Glycogen debranching enzyme	AGL	174.768	1	1	594,540	0.002372545
551	Q6ZR85	Uncharacterized protein C17orf107	C17orf107	19.931	1	1	591,322	0.002359703
552	Q5KU26	Collectin-12	COLEC12	81.517	1	1	590,577	0.00235673
553	P56277	Cx9C motif-containing protein 4	CMC4	7.743	1	1	588,300	0.002347644
554	O15031	Plexin-B2	PLXNB2	205.13	2	2	588,023	0.002346539
555	O43768	Alpha-endosulfine	ENSA	13.39	2	1	583,612	0.002328936
556	Q9UQ35	Serine/arginine repetitive matrix protein 2	SRRM2	299.619	1	1	573,753	0.002289593
557	P09874	Poly [ADP-ribose] polymerase 1	PARP1	113.086	1	1	571,182	0.002279334
558	Q5T9L3	Protein wntless homolog	WLS	62.254	1	1	568,408	0.002268264
559	P11387	DNA topoisomerase 1	TOP1	90.726	1	1	568,088	0.002266987
560	Q96AP7	Endothelial cell-selective adhesion molecule	ESAM	41.176	2	2	567,508	0.002264672
561	P09486	SPARC	SPARC	34.634	1	1	565,893	0.002258228
562	Q9HD20	Endoplasmic reticulum transmembrane helix translocase	ATP13A1	132.957	1	1	561,450	0.002240498
563	O94907	Dickkopf-related protein 1	DKK1	28.669	3	3	559,765	0.002233773
564	Q8TCJ2	Dolichyl-diphosphooligosaccharide--protein glycosyltransferase subunit STT3B	STT3B	93.676	1	1	555,887	0.002218298
565	Q9Y639	Neuroplastin	NPTN	44.388	1	1	555,291	0.00221592

566	O95782	AP-2 complex subunit alpha-1	AP2A1	107.547	1	1	553,322	0.002208062
567	A0A075B6J9	Immunoglobulin lambda variable 2-18	IGLV2-18	12.41	1	1	551,708	0.002201622
568	P25685	DnaJ homolog subfamily B member 1	DNAJB1	38.043	1	1	551,218	0.002199666
569	Q7Z2K6	Endoplasmic reticulum metallopeptidase 1	ERMP1	100.233	1	1	549,939	0.002194562
570	Q9NPE3	H/ACA ribonucleoprotein complex subunit 3	NOP10	7.704	1	1	544,987	0.002174801
571	P07204	Thrombomodulin	THBD	60.329	1	1	542,757	0.002165902
572	O43679;Q86U70	LIM domain-binding protein 2	LDB2	42.794	1	1	542,606	0.0021653
573	Q9Y676	28S ribosomal protein S18b, mitochondrial	MRPS18B	29.396	1	1	539,851	0.002154306
574	Q8NEV8	Exophilin-5	EXPH5	222.524	1	1	539,065	0.002151169
575	P02794	Ferritin heavy chain	FTH1	21.223	6	6	536,355	0.002140355
576	Q92954	Proteoglycan 4	PRG4	151.064	1	1	534,487	0.0021329
577	P13639	Elongation factor 2	EEF2	95.342	5	5	529,478	0.002112912
578	Q9P291	Armadillo repeat-containing X-linked protein 1	ARMCX1	49.179	1	1	520,834	0.002078417
579	Q16204	Coiled-coil domain-containing protein 6	CCDC6	53.291	1	1	515,622	0.002057618
580	P25786	Proteasome subunit alpha type-1	PSMA1	29.556	1	1	515,340	0.002056493
581	O75095	Multiple epidermal growth factor-like domains protein 6	MEGF6	161.19	1	1	512,928	0.002046868
582	Q6ZMI0	Protein phosphatase 1 regulatory subunit 21	PPP1R21	88.315	3	3	508,697	0.002029984
583	Q9Y266	Nuclear migration protein nudC	NUDC	38.244	1	1	505,048	0.002015422
584	Q9NVJ2	ADP-ribosylation factor-like protein 8B	ARL8B	21.538	1	1	502,242	0.002004225
585	P13796	Plastin-2	LCP1	70.291	2	1	502,241	0.002004221
586	A1L4H1	Soluble scavenger receptor cysteine-rich domain-containing protein SSC5D	SSC5D	165.747	1	1	500,935	0.001999009
587	P46087	Probable 28S rRNA (cytosine(4447)-C(5))-methyltransferase	NOP2	89.303	1	1	500,449	0.00199707
588	P26927	Hepatocyte growth factor-like protein	MST1	80.32	3	1	498,276	0.001988398
589	P20742	Pregnancy zone protein	PZP	163.866	5	5	496,038	0.001979467
590	Q96PI1	Small proline-rich protein 4	SPRR4	8.79	1	1	493,752	0.001970345
591	P14923	Junction plakoglobin	JUP	81.745	8	7	493,599	0.001969734
592	Q96KM6	Zinc finger protein 512B	ZNF512B	97.269	1	1	491,678	0.001962069
593	O75475	PC4 and SFRS1-interacting protein	PSIP1	60.104	1	1	487,824	0.001946689
594	P09758	Tumor-associated calcium signal transducer 2	TACSTD2	35.707	1	1	486,391	0.00194097
595	Q9P2S5	WD repeat-containing protein WRAP73	WRAP73	51.587	1	1	485,122	0.001935906
596	O43184	Disintegrin and metalloproteinase domain-containing protein 12	ADAM12	99.545	2	2	484,066	0.001931692
597	P63241;Q6IS14;Q9GZV4	Eukaryotic translation initiation factor 5A-1	EIF5A	16.833	1	1	482,115	0.001923907
598	P48509	CD151 antigen	CD151	28.296	1	1	481,346	0.001920838
599	Q9UBR2	Cathepsin Z	CTSZ	33.869	2	2	481,041	0.001919621
600	Q14118	Dystroglycan 1	DAG1	97.443	2	2	478,503	0.001909493
601	P62491;Q15907	Ras-related protein Rab-11A	RAB11A	24.392	1	1	475,952	0.001899313
602	Q9Y2X3	Nucleolar protein 58	NOP58	59.581	1	1	475,435	0.00189725
603	P16070	CD44 antigen	CD44	81.539	2	2	474,922	0.001895203

604	P37108	Signal recognition particle 14 kDa protein	SRP14	14.572	1	1	473,362	0.001888977
605	P23083	Immunoglobulin heavy variable 1-2	IGHV1-2	13.082	1	1	473,322	0.001888818
606	P02747	Complement C1q subcomponent subunit C	C1QC	25.773	1	1	468,240	0.001868538
607	P46940	Ras GTPase-activating-like protein IQGAP1	IQGAP1	189.254	2	2	464,610	0.001854052
608	P61158	Actin-related protein 3	ACTR3	47.371	1	1	464,105	0.001852037
609	Q9UKV3	Apoptotic chromatin condensation inducer in the nucleus	ACIN1	151.864	1	1	461,327	0.001840951
610	P13667	Protein disulfide-isomerase A4	PDIA4	72.934	2	2	460,551	0.001837854
611	P23528	Cofilin-1	CFL1	18.501	3	2	459,338	0.001833014
612	P46779	60S ribosomal protein L28	RPL28	15.746	1	1	459,182	0.001832391
613	Q92890	Ubiquitin recognition factor in ER-associated degradation protein 1	UFD1	34.5	1	1	453,968	0.001811585
614	Q5D862	Filaggrin-2	FLG2	248.077	1	1	450,944	0.001799517
615	A0A075B6S5;A0A0C4DH67;A0A0C4DH69	Immunoglobulin kappa variable 1-27	IGKV1-27	12.71	1	1	448,883	0.001791293
616	O00115	Deoxyribonuclease-2-alpha	DNASE2	39.581	2	2	446,821	0.001783064
617	P22626	Heterogeneous nuclear ribonucleoproteins A2/B1	HNRNPA2B1	37.428	3	3	446,779	0.001782897
618	P20061	Transcobalamin-1	TCN1	48.206	2	2	442,950	0.001767617
619	P10606	Cytochrome c oxidase subunit 5B, mitochondrial	COX5B	13.695	2	2	439,515	0.001753909
620	P04196	Histidine-rich glycoprotein	HRG	59.578	1	1	439,086	0.001752197
621	P38159;Q96E39	RNA-binding motif protein, X chromosome	RBMX	42.33	1	1	437,645	0.001746447
622	Q9UBC2	Epidermal growth factor receptor substrate 15-like 1	EPS15L1	94.257	2	2	433,137	0.001728457
623	O60687	Sushi repeat-containing protein SRPX2	SRPX2	52.971	1	1	432,791	0.001727077
624	Q6P2E9	Enhancer of mRNA-decapping protein 4	EDC4	151.664	1	1	432,738	0.001726865
625	P26583	High mobility group protein B2	HMGB2	24.032	1	1	431,215	0.001720788
626	Q8N1P7	Beta/gamma crystallin domain-containing protein 2	CRYBG2	177.916	2	2	429,634	0.001714478
627	O43278	Kunitz-type protease inhibitor 1	SPINT1	58.4	2	2	429,274	0.001713042
628	Q8IUX8	Epidermal growth factor-like protein 6	EGFL6	61.318	4	4	428,146	0.001708541
629	P12814	Alpha-actinin-1	ACTN1	103.062	2	1	423,973	0.001691888
630	Q9Y4G6	Talin-2	TLN2	271.62	4	4	423,312	0.00168925
631	P46783	40S ribosomal protein S10	RPS10	18.896	1	1	423,024	0.001688101
632	Q14956	Transmembrane glycoprotein NMB	GPNMB	63.923	3	3	419,094	0.001672418
633	P50238	Cysteine-rich protein 1	CRIP1	8.531	1	1	418,240	0.00166901
634	Q9UJ70	N-acetyl-D-glucosamine kinase	NAGK	37.374	1	1	415,161	0.001656723
635	P08240	Signal recognition particle receptor subunit alpha	SRPRA	69.812	1	1	413,487	0.001650043
636	Q96ST2	Protein IWS1 homolog	IWS1	91.956	2	2	410,533	0.001638255
637	Q02413	Desmoglein-1	DSG1	113.749	3	3	409,644	0.001634707
638	Q00887	Pregnancy-specific beta-1-glycoprotein 9	PSG9	48.272	2	1	406,341	0.001621526
639	P50402	Emerin	EMD	28.994	1	1	406,280	0.001621283
640	Q96DI7	U5 small nuclear ribonucleoprotein 40 kDa protein	SNRNP40	39.31	1	1	406,276	0.001621267
641	Q7Z7K0	COX assembly mitochondrial protein homolog	CMC1	12.488	1	1	402,759	0.001607232

642	P60953	Cell division control protein 42 homolog	CDC42	21.257	1	1	401,933	0.001603936
643	Q14BN4	Sarcolemmal membrane-associated protein	SLMAP	95.199	2	2	401,873	0.001603697
644	P10646	Tissue factor pathway inhibitor	TFPI	35.016	2	2	401,518	0.00160228
645	P10398	Serine/threonine-protein kinase A-Raf	ARAF	67.586	2	2	399,156	0.001592854
646	Q9BWH2	FUN14 domain-containing protein 2	FUNDC2	20.675	1	1	397,654	0.00158686
647	Q8IVF2	Protein AHNAK2	AHNAK2	616.639	1	1	392,142	0.001564865
648	Q4G0J3	La-related protein 7	LARP7	66.898	1	1	389,945	0.001556097
649	P03952	Plasma kallikrein	KLKB1	71.368	1	1	389,500	0.001554321
650	P46379	Large proline-rich protein BAG6	BAG6	119.41	1	1	389,153	0.001552937
651	P62191	26S proteasome regulatory subunit 4	PSMC1	49.185	1	1	385,406	0.001537984
652	P39019	40S ribosomal protein S19	RPS19	16.062	1	1	381,297	0.001521587
653	O75152	Zinc finger CCCH domain-containing protein 11A	ZC3H11A	89.131	3	2	375,240	0.001497416
654	O60318	Germinal-center associated nuclear protein	MCM3AP	218.405	1	1	374,614	0.001494918
655	P09668	Pro-cathepsin H	CTSH	37.392	1	1	374,213	0.001493318
656	Q53RD9	Fibulin-7	FBLN7	47.377	1	1	372,282	0.001485612
657	O43653	Prostate stem cell antigen	PSCA	11.957	1	1	371,697	0.001483278
658	Q06828	Fibromodulin	FMOD	43.179	1	1	368,259	0.001469558
659	Q13753	Laminin subunit gamma-2	LAMC2	130.977	3	3	366,155	0.001461162
660	Q96QR8	Transcriptional activator protein Pur-beta	PURB	33.242	1	1	364,528	0.001454669
661	P01241;P0DML2;P0DML3	Somatotropin	GH1	24.847	2	1	364,354	0.001453975
662	Q8IX12	Cell division cycle and apoptosis regulator protein 1	CCAR1	132.823	3	3	363,100	0.001448971
663	P11142	Heat shock cognate 71 kDa protein	HSPA8	70.899	3	2	362,064	0.001444837
664	Q6UXH9	Inactive serine protease PAMR1	PAMR1	80.199	1	1	359,840	0.001435962
665	Q9NZB2	Constitutive coactivator of PPAR-gamma-like protein 1	FAM120A	121.888	1	1	358,799	0.001431807
666	Q07092	Collagen alpha-1(XVI) chain	COL16A1	157.755	2	2	358,663	0.001431265
667	B2RPK0;P09429	Putative high mobility group protein B1-like 1	HMGB1P1	24.24	1	1	357,471	0.001426508
668	P78504	Protein jagged-1	JAG1	133.801	1	1	357,298	0.001425818
669	P26006	Integrin alpha-3	ITGA3	116.612	1	1	349,805	0.001395916
670	Q6ZNB6	NF-X1-type zinc finger protein NFXL1	NFXL1	101.341	2	2	348,461	0.001390553
671	Q96RW7	Hemicentin-1	HMCN1	613.4	2	2	345,756	0.001379759
672	Q92520	Protein FAM3C	FAM3C	24.68	1	1	345,756	0.001379759
673	P07339	Cathepsin D	CTSD	44.551	2	2	345,394	0.001378314
674	P02511	Alpha-crystallin B chain	CRYAB	20.155	1	1	345,392	0.001378306
675	Q6ZVL8	Putative uncharacterized protein FLJ42384	zzzz	15.892	1	1	345,018	0.001376814
676	Q9C0C9	(E3-independent) E2 ubiquitin-conjugating enzyme	UBE2O	141.296	1	1	344,326	0.001374052
677	A8K2U0	Alpha-2-macroglobulin-like protein 1	A2ML1	161.109	1	1	339,919	0.001356466
678	P29218	Inositol monophosphatase 1	IMPA1	30.188	1	1	339,864	0.001356246
679	Q9Y446	Plakophilin-3	PKP3	87.08	1	1	339,463	0.001354646
680	P02771	Alpha-fetoprotein	AFP	68.678	2	2	336,825	0.001344119
681	Q9NZM1	Myoferlin	MYOF	234.713	2	2	335,960	0.001340667

682	Q8WVM8	Sec1 family domain-containing protein 1	SCFD1	72.381	1	1	334,647	0.001335428
683	Q9Y3A5	Ribosome maturation protein SBDS	SBDS	28.764	2	2	332,982	0.001328783
684	Q86VB7	Scavenger receptor cysteine-rich type 1 protein M130	CD163	125.452	2	2	330,763	0.001319928
685	P68363;Q71U36;Q9BQE3	Tubulin alpha-1B chain	TUBA1B	50.15	1	1	329,628	0.001315399
686	Q05BU3	Putative protein FAM86JP	FAM86JP	4	1	1	329,346	0.001314274
687	Q8IXQ4	GPALPP motifs-containing protein 1	GPALPP1	38.144	1	1	328,310	0.001310139
688	P09871	Complement C1s subcomponent	C1S	76.687	1	1	321,267	0.001282034
689	P07686	Beta-hexosaminidase subunit beta	HEXB	63.112	3	3	320,423	0.001278666
690	P30622	CAP-Gly domain-containing linker protein 1	CLIP1	162.249	1	1	318,591	0.001271355
691	Q5VZK9	F-actin-uncapping protein LRRC16A	CARMIL1	151.557	1	1	317,495	0.001266982
692	P20700	Lamin-B1	LMNB1	66.409	1	1	317,390	0.001266563
693	Q9UHQ9	NADH-cytochrome b5 reductase 1	CYB5R1	34.092	1	1	314,484	0.001254966
694	Q63HQ2	Pikachurin	EGFLAM	111.272	2	2	307,427	0.001226805
695	Q01995	Transgelin	TAGLN	22.61	2	2	306,589	0.001223461
696	P49750	YLP motif-containing protein 1	YLPM1	241.651	1	1	306,302	0.001222315
697	P50552	Vasodilator-stimulated phosphoprotein	VASP	39.83	1	1	304,064	0.001213384
698	O14776	Transcription elongation regulator 1	TCERG1	123.903	1	1	303,282	0.001210264
699	Q13813	Spectrin alpha chain, non-erythrocytic 1	SPTAN1	284.544	2	2	302,562	0.001207391
700	Q6UX06	Olfactomedin-4	OLFM4	57.281	1	1	301,615	0.001203611
701	Q8TCT9	Minor histocompatibility antigen H13	HM13	41.489	1	1	301,191	0.00120192
702	Q13480	GRB2-associated-binding protein 1	GAB1	76.618	1	1	301,069	0.001201433
703	Q92619	Rho GTPase-activating protein 45	ARHGAP45	124.616	1	1	300,097	0.001197554
704	P15692	Vascular endothelial growth factor A	VEGFA	27.045	1	1	299,782	0.001196297
705	O14745	Na(+)/H(+) exchange regulatory cofactor NHE-RF1	SLC9A3R1	38.867	1	1	298,146	0.001189768
706	A0A0B4J1Y8	Immunoglobulin lambda variable 9-49	IGLV9-49	13.022	1	1	297,753	0.0011882
707	P49411	Elongation factor Tu, mitochondrial	TUFM	49.541	1	1	295,198	0.001178004
708	Q9UHB7	AF4/FMR2 family member 4	AFF4	127.461	1	1	294,351	0.001174624
709	P27695	DNA-(apurinic or apyrimidinic site) endonuclease	APEX1	35.555	1	1	293,292	0.001170398
710	P01817	Immunoglobulin heavy variable 2-5	IGHV2-5	13.231	2	2	290,228	0.001158171
711	P49756	RNA-binding protein 25	RBM25	100.186	1	1	289,619	0.001155741
712	Q02952	A-kinase anchor protein 12	AKAP12	191.487	1	1	288,380	0.001150796
713	Q08380	Galectin-3-binding protein	LGALS3BP	65.332	1	1	286,795	0.001144471
714	P05089	Arginase-1	ARG1	34.734	3	3	286,071	0.001141582
715	A0A087WSZ0	Immunoglobulin kappa variable 1D-8	IGKV1D-8	12.835	1	1	284,335	0.001134655
716	Q8WUM0	Nuclear pore complex protein Nup133	NUP133	128.981	1	1	282,277	0.001126442
717	P42566	Epidermal growth factor receptor substrate 15	EPS15	98.659	2	2	281,089	0.001121701
718	Q6ZSR9	Uncharacterized protein FLJ45252	zzzz	37.976	1	1	279,106	0.001113788

719	A0A0B4J1V2	Immunoglobulin heavy variable 2-26	IGHV2-26	13.18	1	1	275,847	0.001100783
720	P31025	Lipocalin-1	LCN1	19.249	2	1	275,617	0.001099865
721	P50570	Dynamin-2	DNM2	98.067	1	1	275,053	0.001097614
722	Q9H3Z4	DnaJ homolog subfamily C member 5	DNAJC5	22.148	1	1	274,923	0.001097096
723	Q16881	Thioredoxin reductase 1, cytoplasmic	TXNRD1	70.876	3	3	272,097	0.001085818
724	Q9H0C8	Integrin-linked kinase-associated serine/threonine phosphatase 2C	ILKAP	42.908	1	1	271,359	0.001082873
725	P83110	Serine protease HTRA3	HTRA3	48.606	1	1	270,442	0.001079214
726	P16157;Q01484;Q12955	Ankyrin-1	ANK1	206.27	2	2	269,464	0.001075311
727	P01591	Immunoglobulin J chain	JCHAIN	18.097	1	1	268,281	0.00107059
728	O60716	Catenin delta-1	CTNND1	108.171	1	1	268,271	0.00107055
729	Q15654	Thyroid receptor-interacting protein 6	TRIP6	50.286	1	1	264,803	0.001056711
730	Q9UQE7	Structural maintenance of chromosomes protein 3	SMC3	141.543	1	1	264,648	0.001056093
731	P07737	Profilin-1	PFN1	15.054	2	2	264,223	0.001054397
732	P30086	Phosphatidylethanolamine-binding protein 1	PEBP1	21.056	2	2	263,607	0.001051938
733	P00492	Hypoxanthine-guanine phosphoribosyltransferase	HPRT1	24.579	2	2	263,542	0.001051679
734	A0A075B6Q5	Immunoglobulin heavy variable 3-64	IGHV3-64	12.888	1	1	262,609	0.001047956
735	Q6ZRV2	Protein FAM83H	FAM83H	127.123	2	2	259,863	0.001036998
736	P54760	Ephrin type-B receptor 4	EPHB4	108.274	1	1	257,318	0.001026842
737	Q9NUL5	Shiftless antiviral inhibitor of ribosomal frameshifting protein	SHFL	33.11	1	1	257,174	0.001026267
738	O00299	Chloride intracellular channel protein 1	CLIC1	26.922	1	1	254,492	0.001015565
739	O75844	CAAX prenyl protease 1 homolog	ZMPSTE24	54.814	1	1	254,406	0.001015221
740	P04259;P48668	Keratin, type II cytoskeletal 6B	KRT6B	60.067	1	1	252,522	0.001007703
741	O75368	SH3 domain-binding glutamic acid-rich-like protein	SH3BGRL	12.772	3	3	252,394	0.001007192
742	Q9BX97	Plasmalemma vesicle-associated protein	PLVAP	50.596	1	1	250,862	0.001001079
743	P17213	Bactericidal permeability-increasing protein	BPI	53.901	2	2	243,756	0.000972722
744	P35030	Trypsin-3	PRSS3	32.532	1	1	242,026	0.000965818
745	P78363	Retinal-specific phospholipid-transporting ATPase ABCA4	ABCA4	255.948	1	1	241,445	0.0009635
746	Q8WUF5	RelA-associated inhibitor	PPP1R13L	89.092	1	1	240,067	0.000958001
747	P84098	60S ribosomal protein L19	RPL19	23.466	1	1	239,986	0.000957678
748	P45973	Chromobox protein homolog 5	CBX5	22.223	1	1	236,677	0.000944473
749	Q9C0C4	Semaphorin-4C	SEMA4C	92.624	1	1	235,122	0.000938267
750	P19823	Inter-alpha-trypsin inhibitor heavy chain H2	ITIH2	106.465	2	2	233,489	0.000931751
751	P49023	Paxillin	PXN	64.507	1	1	233,103	0.000930211
752	O75582	Ribosomal protein S6 kinase alpha-5	RPS6KA5	89.865	1	1	233,072	0.000930087
753	P05107	Integrin beta-2	ITGB2	84.785	1	1	230,352	0.000919233
754	P14866	Heterogeneous nuclear ribonucleoprotein L	HNRNPL	64.135	1	1	226,400	0.000903462
755	P00338	L-lactate dehydrogenase A chain	LDHA	36.688	1	1	224,180	0.000894603
756	Q9Y646	Carboxypeptidase Q	CPQ	51.888	1	1	223,623	0.00089238
757	Q03111	Protein ENL	MLLT1	62.055	1	1	219,938	0.000877675

758	Q92484	Acid sphingomyelinase-like phosphodiesterase 3a	SMPDL3A	51.261	2	2	219,652	0.000876534
759	P18564	Integrin beta-6	ITGB6	85.935	1	1	217,925	0.000869642
760	P15941	Mucin-1	MUC1	122.103	2	2	217,763	0.000868995
761	P15328	Folate receptor alpha	FOLR1	29.82	1	1	214,729	0.000856888
762	P06733	Alpha-enolase	ENO1	47.168	1	1	214,676	0.000856677
763	Q12860	Contactin-1	CNTN1	113.322	1	1	213,278	0.000851098
764	P10619	Lysosomal protective protein	CTSA	54.465	1	1	212,724	0.000848887
765	P09382	Galectin-1	LGALS1	14.713	1	1	211,674	0.000844697
766	Q9NQ36	Signal peptide, CUB and EGF-like domain-containing protein 2	SCUBE2	109.957	1	1	211,285	0.000843145
767	P04075	Fructose-bisphosphate aldolase A	ALDOA	39.418	4	4	210,174	0.000838711
768	P30405	Peptidyl-prolyl cis-trans isomerase F, mitochondrial	PPIF	22.04	1	1	210,086	0.00083836
769	Q9BRJ2	39S ribosomal protein L45, mitochondrial	MRPL45	35.352	1	1	207,606	0.000828463
770	Q96PK6	RNA-binding protein 14	RBM14	69.491	1	1	204,782	0.000817194
771	P07195	L-lactate dehydrogenase B chain	LDHB	36.639	1	1	203,949	0.00081387
772	A0A0U1RQF7	DPEP2 neighbor protein	DPEP2NB	13.4	1	1	203,825	0.000813375
773	Q15059	Bromodomain-containing protein 3	BRD3	79.544	1	1	199,182	0.000794847
774	P57737	Coronin-7	CORO7	100.607	1	1	198,813	0.000793374
775	O14974	Protein phosphatase 1 regulatory subunit 12A	PPP1R12A	115.284	1	1	197,043	0.000786311
776	O95183	Vesicle-associated membrane protein 5	VAMP5	12.803	1	1	193,724	0.000773066
777	Q9H2D6	TRIO and F-actin-binding protein	TRIOBP	261.381	3	3	193,082	0.000770505
778	O75420	GRB10-interacting GYF protein 1	GIGYF1	114.601	1	1	191,603	0.000764602
779	Q8TE58;Q9UHI8	A disintegrin and metalloproteinase with thrombospondin motifs 15	ADAMTS15	103.288	1	1	190,587	0.000760548
780	Q9Y3R0	Glutamate receptor-interacting protein 1	GRIP1	122.425	1	1	189,945	0.000757986
781	Q9H4A3	Serine/threonine-protein kinase WNK1	WNK1	250.797	1	1	187,590	0.000748588
782	P15311;P26038;P35241	Ezrin	EZR	69.414	1	1	185,200	0.000739051
783	P31944	Caspase-14	CASP14	27.681	1	1	183,367	0.000731736
784	P98179	RNA-binding protein 3	RBM3	17.171	1	1	181,940	0.000726042
785	Q9Y4L1	Hypoxia up-regulated protein 1	HYOU1	111.34	1	1	180,511	0.000720339
786	P61247	40S ribosomal protein S3a	RPS3A	29.947	1	1	180,481	0.00072022
787	Q9BY42	Replication termination factor 2	RTF2	33.889	1	1	179,389	0.000715862
788	A0A1B0GUS4;P68036	Ubiquitin-conjugating enzyme E2 L5	UBE2L5	17.876	1	1	177,748	0.000709313
789	Q86UP2	Kinectin	KTN1	156.279	2	2	177,537	0.000708471
790	P60842;Q14240	Eukaryotic initiation factor 4A-I	EIF4A1	46.156	1	1	175,387	0.000699892
791	P26639	Threonine--tRNA ligase 1, cytoplasmic	TARS1	83.436	2	1	171,430	0.000684101
792	P53420	Collagen alpha-4(IV) chain	COL4A4	164.039	1	1	170,528	0.000680502
793	Q01082	Spectrin beta chain, non-erythrocytic 1	SPTBN1	274.614	1	1	170,370	0.000679871
794	P26641	Elongation factor 1-gamma	EEF1G	50.12	1	1	169,176	0.000675106
795	Q9BTU6	Phosphatidylinositol 4-kinase type 2-alpha	PI4K2A	54.021	1	1	168,484	0.000672345
796	Q92542	Nicastrin	NCSTN	78.411	1	1	166,287	0.000663578
797	P61313	60S ribosomal protein L15	RPL15	24.145	1	1	164,651	0.000657049
798	Q8WWM7	Ataxin-2-like protein	ATXN2L	113.376	1	1	163,712	0.000653302

799	Q8N8U9	BMP-binding endothelial regulator protein	BMPER	75.999	1	1	161,309	0.000643713
800	Q8IWA5	Choline transporter-like protein 2	SLC44A2	80.125	2	2	159,078	0.00063481
801	P02746	Complement C1q subcomponent subunit B	C1QB	26.72	1	1	157,437	0.000628261
802	Q9H0W9	Ester hydrolase C11orf54	C11orf54	35.114	1	1	155,569	0.000620807
803	P62258	14-3-3 protein epsilon	YWHAE	29.172	2	1	154,073	0.000614837
804	Q9HDC9	Adipocyte plasma membrane-associated protein	APMAP	46.48	1	1	154,035	0.000614685
805	P01861	Immunoglobulin heavy constant gamma 4	IGHG4	35.941	1	1	153,817	0.000613815
806	P26012	Integrin beta-8	ITGB8	85.633	1	1	150,083	0.000598915
807	P07602	Prosaposin	PSAP	58.113	2	2	148,920	0.000594274
808	P08311	Cathepsin G	CTSG	28.836	1	1	148,579	0.000592913
809	Q8IX30	Signal peptide, CUB and EGF-like domain-containing protein 3	SCUBE3	109.285	1	1	144,559	0.000576871
810	Q08188	Protein-glutamine gamma-glutamyltransferase E	TGM3	76.631	1	1	144,505	0.000576655
811	P27816	Microtubule-associated protein 4	MAP4	121.007	2	2	143,910	0.000574281
812	P19338	Nucleolin	NCL	76.615	1	1	143,869	0.000574117
813	P62316	Small nuclear ribonucleoprotein Sm D2	SNRPD2	13.525	1	1	142,225	0.000567557
814	P52758	2-iminobutanoate/2-iminopropanoate deaminase	RIDA	14.494	1	1	141,087	0.000563016
815	Q8N1N4	Keratin, type II cytoskeletal 78	KRT78	56.868	2	2	138,951	0.000554492
816	P54725	UV excision repair protein RAD23 homolog A	RAD23A	39.609	1	1	136,929	0.000546423
817	P19827	Inter-alpha-trypsin inhibitor heavy chain H1	ITIH1	101.39	2	2	136,069	0.000542991
818	P14618	Pyruvate kinase PKM	PKM	57.938	2	2	134,840	0.000538087
819	Q86YZ3	Hornerin	HRNR	282.391	1	1	131,196	0.000523545
820	P12277	Creatine kinase B-type	CKB	42.646	2	2	130,277	0.000519878
821	P05141	ADP/ATP translocase 2	SLC25A5	32.855	2	1	129,140	0.00051534
822	Q12906	Interleukin enhancer-binding factor 3	ILF3	95.341	1	1	127,698	0.000509586
823	P01718	Immunoglobulin lambda variable 3-27	IGLV3-27	12.163	1	1	127,154	0.000507415
824	Q9Y4F1	FERM, ARHGEF and pleckstrin domain-containing protein 1	FARP1	118.635	1	1	126,700	0.000505603
825	Q9UKX7	Nuclear pore complex protein Nup50	NUP50	50.145	1	1	124,112	0.000495276
826	O60361;P15531;P22392	Putative nucleoside diphosphate kinase	NME2P1	15.528	1	1	123,278	0.000491948
827	Q86YQ8	Copine-8	CPNE8	63.108	2	1	120,297	0.000480052
828	P02549	Spectrin alpha chain, erythrocytic 1	SPTA1	280.019	1	1	119,554	0.000477087
829	Q15942	Zyxin	ZYX	61.277	1	1	117,073	0.000467186
830	A0A0C4DH33	Immunoglobulin heavy variable 1-24	IGHV1-24	12.824	1	1	116,146	0.000463487
831	Q13976	cGMP-dependent protein kinase 1	PRKG1	76.365	1	1	115,560	0.000461149
832	O14786	Neuropilin-1	NRP1	103.136	2	2	113,948	0.000454716
833	Q9UKP4	A disintegrin and metalloproteinase with thrombospondin motifs 7	ADAMTS7	184.098	1	1	113,158	0.000451563
834	Q03252	Lamin-B2	LMNB2	69.949	1	1	110,019	0.000439037
835	Q7KZF4	Staphylococcal nuclease domain-containing protein 1	SND1	101.999	1	1	107,026	0.000427093
836	P14555	Phospholipase A2, membrane associated	PLA2G2A	16.084	1	1	103,920	0.000414699

837	O95971	CD160 antigen	CD160	19.807	1	1	103,819	0.000414296
838	Q04206	Transcription factor p65	RELA	60.219	1	1	103,804	0.000414236
839	Q96AE4	Far upstream element-binding protein 1	FUBP1	67.561	1	1	95,356	0.000380525
840	P11274	Breakpoint cluster region protein	BCR	142.823	1	1	92,852	0.00037053
841	P55209	Nucleosome assembly protein 1-like 1	NAP1L1	45.374	1	1	86,137	0.000343733
842	O15014	Zinc finger protein 609	ZNF609	151.193	1	1	85,152	0.000339804
843	P01833	Polymeric immunoglobulin receptor	PIGR	83.284	1	1	83,561	0.000333455
844	Q14103	Heterogeneous nuclear ribonucleoprotein D0	HNRNPD	38.435	1	1	83,207	0.000332044
845	O95297	Myelin protein zero-like protein 1	MPZL1	29.082	1	1	80,806	0.00032246
846	A0AVI4	E3 ubiquitin-protein ligase TM129	TMEM129	40.464	1	1	80,533	0.000321372
847	Q5T749	Keratinocyte proline-rich protein	KPRP	64.137	1	1	80,038	0.000319395
848	P14854	Cytochrome c oxidase subunit 6B1	COX6B1	10.192	1	1	77,686	0.000310011
849	Q96FQ6	Protein S100-A16	S100A16	11.802	1	1	76,167	0.00030395
850	Q9BZE9	Tether containing UBX domain for GLUT4	ASPSCR1	60.182	1	1	75,137	0.000299837
851	Q96EK7	Constitutive coactivator of peroxisome proliferator-activated receptor gamma	FAM120B	103.785	1	1	74,440	0.000297055
852	Q9NXV6	CDKN2A-interacting protein	CDKN2AIP	61.126	1	1	73,705	0.000294126
853	Q8NFQ8	Torsin-1A-interacting protein 2	TOR1AIP2	51.264	1	1	72,894	0.000290886
854	P49454	Centromere protein F	CENPF	357.532	1	1	72,765	0.000290374
855	P63220	40S ribosomal protein S21	RPS21	9.109	1	1	72,121	0.000287801
856	O75084;Q14332	Frizzled-7	FZD7	63.621	1	1	71,725	0.000286221
857	P23396	40S ribosomal protein S3	RPS3	26.684	1	1	71,705	0.000286142
858	Q9BY44	Eukaryotic translation initiation factor 2A	EIF2A	64.99	1	1	70,866	0.000282794
859	Q99497	Parkinson disease protein 7	PARK7	19.892	1	1	70,691	0.000282096
860	P62081	40S ribosomal protein S7	RPS7	22.126	1	1	69,798	0.000278531
861	P62318	Small nuclear ribonucleoprotein Sm D3	SNRPD3	13.915	1	1	68,714	0.000274208
862	Q9HD45	Transmembrane 9 superfamily member 3	TM9SF3	67.89	1	1	59,140	0.000236001
863	Q9HCY8	Protein S100-A14	S100A14	11.661	1	1	58,343	0.000232819
864	P61204;P84077;P84085	ADP-ribosylation factor 3	ARF3	20.598	1	1	55,484	0.000221413
865	P07477;P07478;Q8NHM4	Trypsin-1	PRSS1	26.559	1	1	54,062	0.000215737
866	Q16853	Membrane primary amine oxidase	AOC3	84.621	1	1	53,232	0.000212423
867	Q04695	Keratin, type I cytoskeletal 17	KRT17	48.106	2	2	50,561	0.000201765
868	P15586	N-acetylglucosamine-6-sulfatase	GNS	62.083	1	1	50,362	0.000200972
869	P30048	Thioredoxin-dependent peroxide reductase, mitochondrial	PRDX3	27.693	1	1	47,408	0.000189182
870	Q9H7B2	Ribosome production factor 2 homolog	RPF2	35.585	1	1	45,011	0.000179618
871	Q9UMD9	Collagen alpha-1(XVII) chain	COL17A1	150.42	1	1	41,211	0.000164454
872	P33993	DNA replication licensing factor MCM7	MCM7	81.31	1	1	40,913	0.000163266
873	P10809	60 kDa heat shock protein, mitochondrial	HSPD1	61.054	1	1	39,833	0.000158955
874	A0A0J9YW62	IGv domain-containing protein (Fragment)	zzzz	13.249	1	1	39,192	0.000156399
875	Q00610	Clathrin heavy chain 1	CLTC	191.617	1	1	33,378	0.000133197
						Sum	25,059,165,743	100

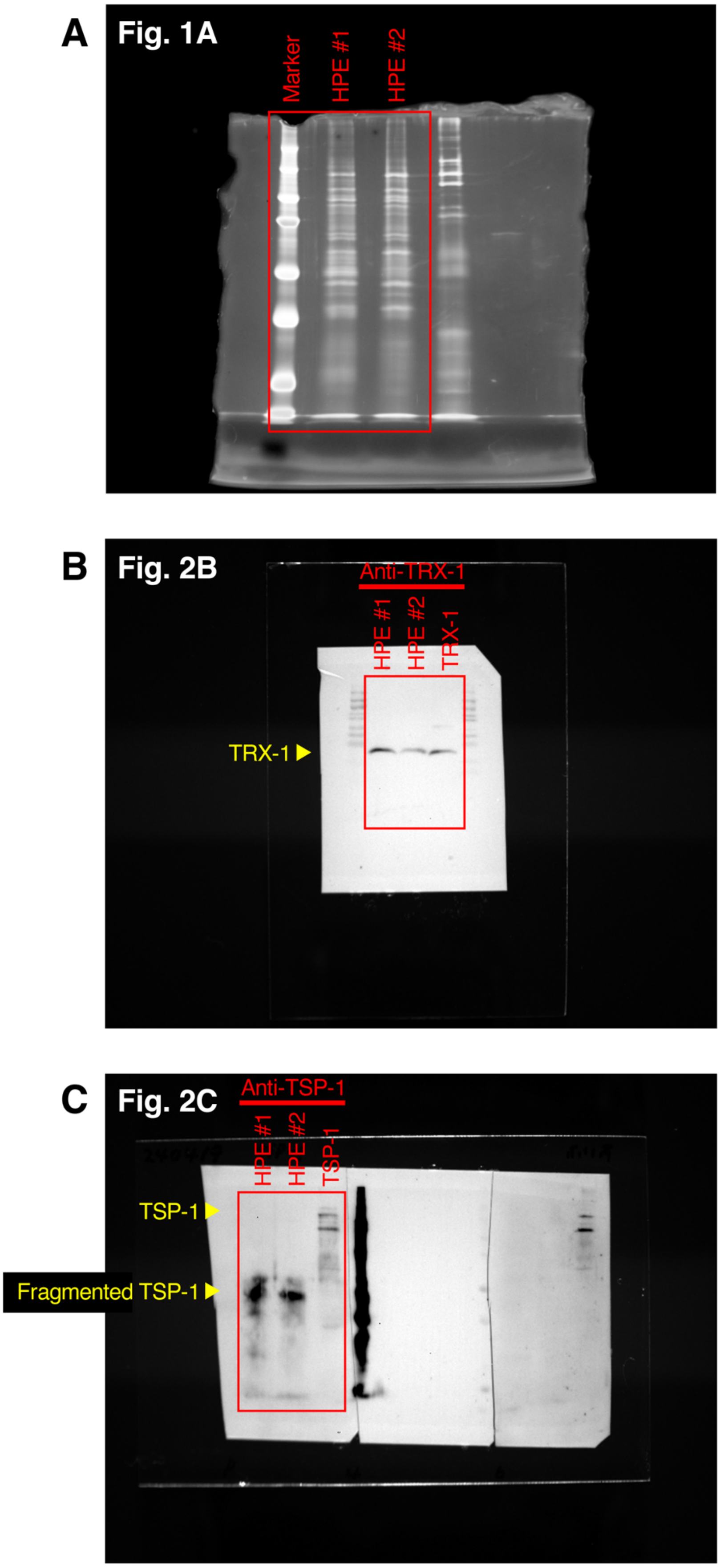

Supplementary Figure S1. Full-length blots/gels of Fig. 1A (A), 2B (B) and 2C (C).

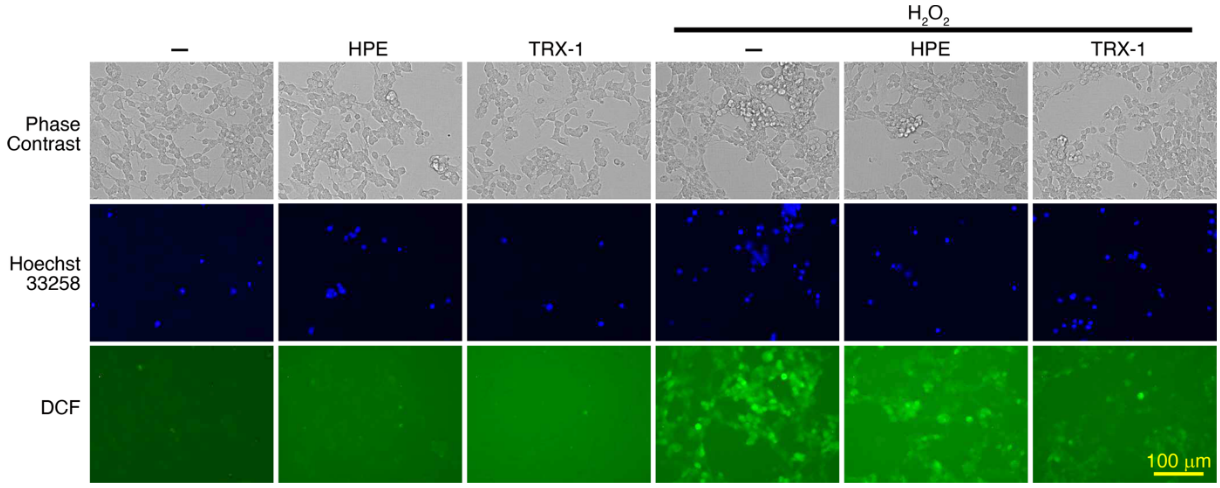

Supplementary Figure S2. Larger images of Fig. 3A.
